# Enumeration of binary trees compatible with a perfect phylogeny

**DOI:** 10.1007/s00285-022-01748-w

**Published:** 2022-05-12

**Authors:** Julia A. Palacios, Anand Bhaskar, Filippo Disanto, Noah A. Rosenberg

**Affiliations:** 1 Department of Statistics, Stanford University, Stanford, CA USA; 2 Department of Biomedical Data Science, Stanford Medicine, Stanford, CA USA; 3 Department of Genetics, Stanford University, Stanford, CA USA; 4 Department of Mathematics, University of Pisa, Pisa, Italy; 5 Department of Biology, Stanford University, Stanford, CA USA

**Keywords:** 05C05, 92D15

## Abstract

Evolutionary models used for describing molecular sequence variation suppose that at a non-recombining genomic segment, sequences share ancestry that can be represented as a genealogy—a rooted, binary, timed tree, with tips corresponding to individual sequences. Under the infinitely-many-sites mutation model, mutations are randomly superimposed along the branches of the genealogy, so that every mutation occurs at a chromosomal site that has not previously mutated; if a mutation occurs at an interior branch, then all individuals descending from that branch carry the mutation. The implication is that observed patterns of molecular variation from this model impose combinatorial constraints on the hidden state space of genealogies. In particular, observed molecular variation can be represented in the form of a perfect phylogeny, a tree structure that fully encodes the mutational differences among sequences. For a sample of *n* sequences, a perfect phylogeny might not possess *n* distinct leaves, and hence might be compatible with many possible binary tree structures that could describe the evolutionary relationships among the *n* sequences. Here, we investigate enumerative properties of the set of binary ranked and unranked tree shapes that are compatible with a perfect phylogeny, and hence, the binary ranked and unranked tree shapes conditioned on an observed pattern of mutations under the infinitely-many-sites mutation model. We provide a recursive enumeration of these shapes. We consider both perfect phylogenies that can be represented as binary and those that are multifurcating. The results have implications for computational aspects of the statistical inference of evolutionary parameters that underlie sets of molecular sequences.

## Introduction

Coalescent and mutation models are used in population genetics to estimate evolutionary parameters from samples of molecular sequences (Marjoram and Tavaré 2006). The central idea is that observed molecular variation is the result of a process of mutation along the branches of the genealogy of the sample. This genealogy is a timed tree that represents the ancestral relationships of the sample at a chromosomal segment. Consisting of a tree topology and its branch lengths, the genealogy is a nuisance parameter that is modeled as a realization of the coalescent process dictated by evolutionary parameters—which are in turn inferred by integrating over the space of genealogies. For large sample sizes, however, this integration is computationally challenging because the state space of tree topologies increases exponentially with the number of sampled sequences.

Recently, a coarser coalescent model known as the *Tajima coalescent* (Tajima 1983; Sainudiin et al. 2015), coupled with the infinitely-many-sites mutation model (Kimura 1969), has been introduced for population-genetic inference problems (Palacios et al. 2019). Whereas the standard coalescent model (Kingman 1982) induces a probability measure on the space of ranked labeled tree topologies, the Tajima coalescent induces a probability measure on the space of ranked *unlabeled* tree topologies. Removing the labels of the tips from the tree topology, as in the Tajima coalescent, reduces the cardinality of the space of tree topologies substantially, shrinking computation time in inference problems.

Under infinitely-many-sites mutation, only a subset of tree topologies (labeled or unlabeled) are compatible with an observed data set, so that the computational complexity of inference varies among different data sets. Hence, Cappello et al. (2020a) used importance sampling to approximate cardinalities of the spaces of labeled and unlabeled ranked tree shapes conditioned on a data set of molecular sequences, demonstrating a striking reduction of the cardinality of the space of ranked unlabeled tree shapes versus the labeled counterpart when conditioning on observed data with a sparse number of mutations. Here, we extend beyond the approximate work of Cappello et al. (2020a) and obtain exact results. We provide a recursive algorithm for exact computation of the cardinality of the spaces of labeled and unlabeled ranked tree shapes compatible with a sequence data set. We provide a number of other enumerative results relevant for inference of tree topologies in phylogenetics and population genetics. Python code for enumeration is available at https://colab.research.google.com/drive/1cAx2xyn7OtmG-F-9nxJ3CHRc7e7AjuCj?usp=sharing.

## Preliminaries

### Types of trees

**Fig. 1:**
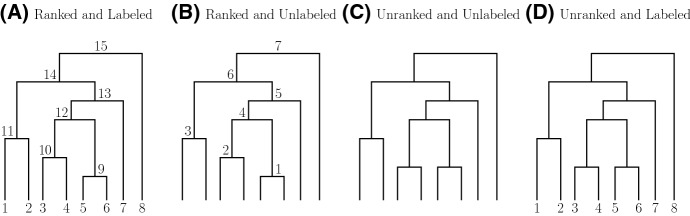
Different types of trees. **A** A ranked labeled tree shape. **B** A ranked unlabeled tree shape. **C** An unranked unlabeled tree shape. **D** An unranked labeled tree shape. The ranked unlabeled tree shape in (**B**) is obtained by discarding leaf labels from the ranked labeled tree shape in (**A**). The unranked labeled tree shape in (**D**) is obtained by discarding the sequence of internal node ranks in (**A**). The unranked unlabeled tree shape in (**C**) is obtained by discarding the sequence of internal node ranks in (**B**) or the leaf labels in (**D**)

The **coalescent** is a continuous-time Markov chain with values in the space ${\mathcal {P}}_{n}$ of partitions of $[n]=\{1,2,\ldots ,n\}$ (Kingman 1982). The process starts with the trivial partition of *n* singletons, labeled $\{1\},\{2\},\ldots ,\{n\}$, at time 0; at each transition, two blocks are chosen uniformly at random to merge into a single block. The process ends with a single block with label $\{1,2,\ldots ,n\}$. In the standard coalescent, the holding times are exponentially distributed with rate $$\left( {\begin{array}{c}k\\ 2\end{array}}\right) $$ when there are *k* blocks. Transition probabilities for the coalescent can be factored into two independent components, a pure death process and a discrete jump chain. A full realization of the process can be represented by a timed rooted binary tree: a genealogy. The tips of the genealogy are labeled by $\{1,2,\ldots ,n\}$. Figure 1A shows a realization of the jump process, a ranked labeled tree shape.

A lumping of the standard coalescent process, called the **Tajima coalescent** (Sainudiin et al. 2015), consists in removing the labels of the tips of the genealogy. The pure death process of the lumped process is the same as the standard coalescent. The discrete jump chain can be described as a simple urn process (Janson and Kersting 2011). Start with an urn of *n* balls labeled 0; at the *i*th transition, draw two balls and return one to the urn with label *i*. The process ends when there is a single ball with label $n-1$ in the urn. A full realization of the urn process can be represented as a ranked unlabeled tree shape with internal nodes labeled by the transition index.

A **ranked labeled tree shape** of size *n*, denoted by $T^{L}_{n}$, is a rooted binary labeled tree of *n* leaves with a total ordering for the internal nodes. Without loss of generality, we use label set [*n*] to label the *n* leaves. The space of ranked labeled tree shapes with *n* leaves will be denoted by ${\mathcal {T}}^{L}_{n}$. Figure 1A shows an example of a ranked labeled tree shape with $n=8$ leaves. Ranked labeled tree shapes are also known as labeled histories.

**Fig. 2:**
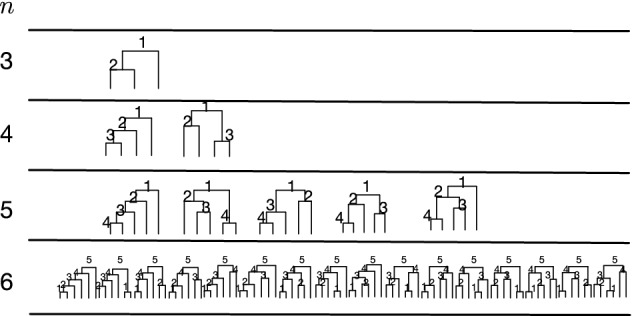
An enumeration of all possible ranked tree shapes with 3, 4, 5, and 6 leaves

A **ranked unlabeled tree shape** of size *n*, denoted by $T^{R}_{n}$, is a rooted binary unlabeled tree of *n* leaves with a total ordering for the internal nodes. The space of ranked unlabeled tree shapes with *n* leaves will be denoted by ${\mathcal {T}}^{R}_{n}$. Figure 1B shows an example of a ranked unlabeled tree shape with $n=8$ leaves. We will refer to a ranked unlabeled tree shape simply as a ranked tree shape; these ranked tree shapes are also known as unlabeled histories, or Tajima trees. Figure 2 shows all ranked unlabeled tree shapes with 3, 4, 5,  and 6 leaves.

An **unranked unlabeled tree shape** of size *n*, denoted by $T_{n}$, is a rooted binary unlabeled tree of *n* leaves with unlabeled internal nodes. The space of unranked (unlabeled) tree shapes with *n* leaves will be denoted by ${\mathcal {T}}_{n}$. Figure 1C shows an example of an unranked unlabeled tree shape with $n=8$ leaves. These shapes are also called unlabeled topologies or Otter trees (Otter 1948).

An **unranked labeled tree shape** of size *n*, denoted by $T^X_n$, is a rooted binary labeled tree of *n* leaves with unlabeled internal nodes. The space of unranked labeled tree shapes with *n* leaves will be denoted by ${\mathcal {T}}^X_{n}$. Figure 1D shows an example of an unranked labeled tree shape with $n=8$ leaves. These tree shapes are also called labeled topologies.

### Mutations on trees

Many generative models of neutral molecular evolution assume that a process of mutation is superimposed on the genealogy as a continuous-time Markov process. In the **infinitely-many-sites mutation model**, every mutation along the branches of the tree occurs at a chromosomal site that has not previously mutated (Kimura 1969). Therefore, if a mutation occurs at an interior branch along the genealogy, all sequences descended from that branch carry the mutation. Because every site can mutate at most once, the sequence of mutated sites can be encoded as a binary sequence, with 0 denoting the ancestral type and 1 denoting the mutant type at any site; we assume that the ancestral type is known, and that it is denoted by 0.

**Fig. 3:**
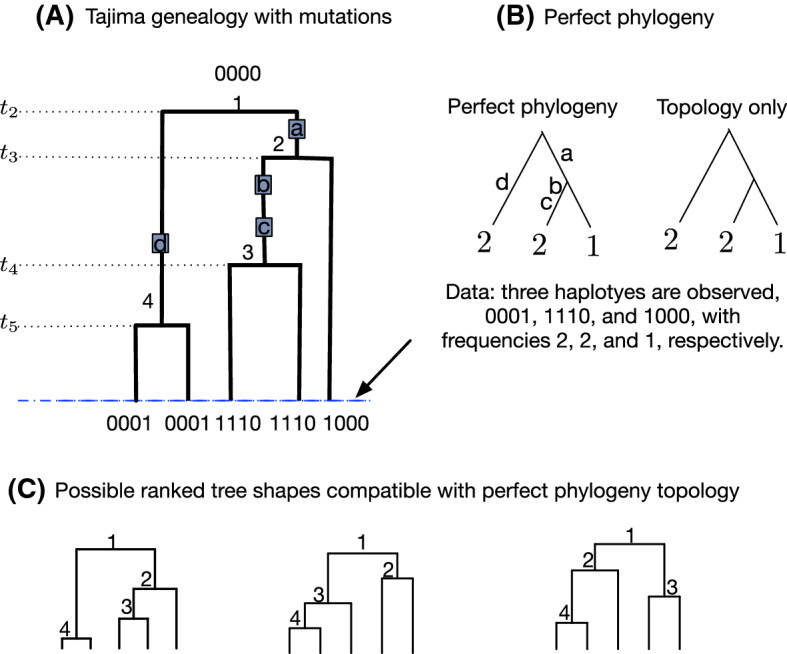
Tajima coalescent and infinitely-many-sites generative model of binary molecular data. **A** A Tajima genealogy of 5 individuals, with 4 superimposed mutations depicted as gray squares. The root is labeled by the ancestral type 0000, and the leaves are labeled by the genetic type at each of three mutated sites. The first two leaves from left to right are labeled 0001 because one mutation occurs in their path to the root. The third and fourth individuals have three mutations in their path to the root and are labeled 1110; the last individual is labeled 1000 because only one mutation occurs along its path to the root. The order and label of the mutations is unimportant; however, it is assumed that the same position, or site, in a sequence of 0s and 1s corresponds across individuals. For ease of exposition, we label the mutations a, b, c and d. The first site corresponds to mutation a, the second to b, the third to c, and the fourth to d. **B** Left, a perfect phylogeny representation of the observed data at the tips of (**A**). Data consist of 3 unique haplotypes 0001, 1110 and 1000, with frequencies 2, 2, and 1. The corresponding frequencies are the labels of tips of the perfect phylogeny. Right, perfect phylogeny topology obtained by removing the edge labels of the perfect phylogeny. **C** The only three ranked tree shapes compatible with the perfect phylogeny topology in (**B**)

Figure 3A shows a realization of the Tajima coalescent together with a realization of mutations from the infinitely-many-sites mutation model with 5 individuals and 4 mutated sites. In what follows, we assume that we observe molecular data only as binary sequences at the tips of the tree.

### Observed binary molecular sequence data as a perfect phylogeny

The **perfect phylogeny algorithm**, proposed by Gusfield (1991), generates a graphical representation of binary molecular sequence data that have been produced according to the infinitely-many-sites mutation model. Label individual sequences $1,2, \ldots , n$, and label mutated or “segregating” sites $a,b,\ldots$. The original algorithm generates a rooted tree structure known as a **perfect phylogeny**, with tips labeled $1,2,\ldots ,n$ and with edges labeled $a,b,\ldots$, that is in bijection with the observed “labeled data.” An edge can have no labels, one label, or more than one label. Perfect phylogenies have been central to coalescent-based inference algorithms, in which maximum likelihood or Bayesian estimation of evolutionary parameters that have given rise to the particular distribution of mutations and clade sizes on the perfect phylogeny are sought by importance sampling or Markov chain Monte Carlo (Griffiths and Tavaré 1994; Stephens and Donnelly 2000; Palacios et al. 2019; Cappello et al. 2020b).

In this study, we assume that individual sequences are not uniquely labeled, but instead, are identified by their sequences of 0s and 1s, or **haplotypes**. Hence, the number of tips in our perfect phylogeny is the number of unique haplotypes, and the labels at the tips correspond to the observed frequencies of the haplotypes. For the genealogy in Fig. 3A and B shows the perfect phylogeny of the data observed at its tips.

The key assumption of the bijection between sequence data sets and perfect phylogenies is that if a site mutates once, then all descendants of the lineage on which the mutation occurred must also have the mutation—and no other individuals will have the mutation. That is, every unique mutation, or site, partitions the sample of haplotypes into two groups: those with the mutation and those without the mutation. Hence, we group sites that induce the same partition on the haplotypes, and we call each such group of sites a **mutation group**.

In this study, we are not concerned with the mutation labels, and hence, we remove the edge labels of the perfect phylogeny (right side of Fig. 3B), so that we consider only the topology of the perfect phylogeny. In dropping the edge labels, we treat a perfect phylogeny topology as a perfect phylogeny. Henceforth, a **perfect phylogeny** is a multifurcating rooted tree with *k* leaves, representing *k* distinct haplotypes, each labeled by a positive integer $(n_{i})_{1\le i \le k}$, with $\sum ^{k}_{i=1}n_{i}=n$. We use the symbol $\Pi _{n}$ to denote the space of perfect phylogenies of size *n* sequences, and we use $\pi \in \Pi _{n}$ to denote a perfect phylogeny with *n* sequences.

A perfect phylogeny $\pi$ is completely specified in a parenthetical notation, in which every leaf is represented by its label, every binary internal node is represented by $(\cdot ,\cdot )$, and every multifurcating internal node is represented by $(\cdot ,\ldots ,\cdot )$. For example, the perfect phylogeny $\pi _{1}$ on the right in Fig. 3B in parenthetical notation can be written (2, (2, 1)) or ((2, 1), 2), indicating that there are two internal nodes, one merging leaves (2, 1) and one merging (2, 1) with 2.

The most extreme unresolved perfect phylogeny with *n* tips—the perfect phylogeny that is compatible with all ranked tree shapes with *n* tips—has two representations. It can be written as a star, in which the root has degree *n* and is the only internal node, that is, $\pi =(1,1,\ldots ,1)$. It can also be written as a single node $\pi =(n)$. For our purposes, with mutations discarded, the star and single-node perfect phylogenies are indistinguishable, and they will be represented as a single-node perfect phylogeny. Details of the algorithm for generating the perfect phylogeny from binary molecular data can be found in Cappello et al. (2020a), which presents a slight modification to Gusfield’s algorithm (Gusfield 1991).

We say that a binary tree *T* is **compatible** with a perfect phylogeny $\pi$ if the tree can be reduced to $\pi$ by collapsing internal edges of *T*. The number of tree shapes, ranked or unranked, that are compatible with a perfect phylogeny gives the cardinality of the corresponding posterior sampling tree space in statistical inference from sequence data sets. Given a perfect phylogeny $\pi \in \Pi _{n}$, we are interested in calculating the number of compatible ranked tree shapes with *n* leaves and the number of compatible unranked tree shapes with *n* leaves.

### Known enumerative results

In advance of our effort to count tree shapes compatible with a perfect phylogeny, we state some known enumerative results for the unconstrained spaces of ranked labeled tree shapes, unranked labeled tree shapes, ranked unlabeled tree shapes, and unranked unlabeled tree shapes (Steel 2016).

Let $L_{n}=|{\mathcal {T}}^{L}_{n}|$ denote the cardinality of the space of ranked labeled trees with *n* leaves. Then

1 $$\begin{aligned} L_{n}=\prod ^{n}_{i=2}\left( {\begin{array}{c}i\\ 2\end{array}}\right) =\frac{n!(n-1)!}{2^{n-1}}. \end{aligned}$$

The product is obtained by noting that for each decreasing *i* from *n* to 2, there are $$\left( {\begin{array}{c}i\\ 2\end{array}}\right) $$ ways of merging two labeled branches. The sequence of values of $L_n$ begins 1, 1, 3, 18, 180, 2700, 56,700.

Let $X_n=|{\mathcal {T}}^{X}_{n}|$ denote the number of unranked labeled trees with *n* leaves. We have

2 $$\begin{aligned} X_{n}=(2n-3)!! = \frac{(2n-2)!}{2^{n-1} (n-1)!}. \end{aligned}$$

To generate trees in ${\mathcal {T}}^{X}_{n}$ from trees in ${\mathcal {T}}^{X}_{n-1}$, a pendant edge connected to the *n*th label can be placed along each of the $2n-3$ edges of a tree with $n-1$ leaves, including an edge above the root. $X_n$ is obtained as the solution to the recursion $X_n = (2n-3)X_{n-1}$, with $X_1=1$. The sequence of values of $X_n$ begins 1, 1, 3, 15, 105, 945, 10,395.

The number of ranked tree shapes with *n* tips is the $(n-1)$-th Euler zigzag number (Stanley 2012). Let $R_{n}=|{\mathcal {T}}^{R}_{n}|$ denote the number of ranked tree shapes with *n* leaves. We have the following recursion:

3 $$\begin{aligned} R_1&= 1, \, R_2= 1, \nonumber \\ R_{n+1}&= \frac{1}{2} \sum _{k=0}^{n-1} {n-1 \atopwithdelims ()k} R_{k+1} R_{n-k}, \, n \ge 2. \end{aligned}$$

The sequence of values of $R_n$ begins 1, 1, 1, 2, 5, 16, 61. For $n \ge 1$, if the tree has $n+1$ tips, and hence *n* interior nodes, then the root divides the tree into two ranked subtrees $T^{R}_1$ and $T^{R}_2$, where $T^{R}_1$ has *k* interior nodes, $0 \le k \le n-1$, and $T^{R}_2$ has $n-1 - k$ interior nodes. There are ${n-1 \atopwithdelims ()k}$ ways of interleaving the *k* and $n-1-k$ interior nodes of $T^{R}_1$ and $T^{R}_2$, such that the relative orderings of the interior nodes of $T^{R}_1$ and $T^{R}_2$ are preserved in the interleaving. The number of possible ranked tree shapes with such a configuration is ${n-1 \atopwithdelims ()k} R_{k+1} R_{n-k}$. Summing over the possibilities for *k* from 0 to $n-1$, and acknowledging that the identity of $T^{R}_1$ and $T^{R}_2$ can be interchanged, we get Eq. 3.

Let $S_{n}=|{\mathcal {T}}_{n}|$ denote the number of unranked tree shapes with *n* leaves. We have the following recursion:

4 $$\begin{aligned} S_1&= 1, \nonumber \\ S_{2n-1}&= \sum ^{n-1}_{k=1}S_{k}S_{2n-1-k}, \quad n \ge 2, \end{aligned}$$

5 $$\begin{aligned} S_{2n}&= \bigg (\sum ^{n-1}_{k=1}S_{k}S_{2n-k}\bigg )+\frac{1}{2}S_{n}(S_{n}+1), \quad n \ge 1. \end{aligned}$$

$S_{n}$ is the *n*th Wedderburn–Etherington number (Harding 1971). The sequence begins 1, 1, 1, 2, 3, 6, 11. When the number of leaves is $2n-1$, the root divides the tree shape into two subtree shapes $T_{1}$ and $T_{2}$ with *k* and $2n-1-k$ leaves, for $k=1,2,\ldots ,n-1$. When the number of leaves is even, the root divides the tree shape into subtree shapes with *k* and $2n-k$ leaves for $k=1,2,\ldots ,n-1$ or two subtree shapes with *n* leaves; these tree shapes are indistinguishable in $S_{n}$ cases and distinguishable in $\frac{1}{2}S_n(S_{n}-1)$ cases.

## Enumeration for binary perfect phylogenies

To count ranked and unranked tree shapes compatible with a perfect phylogeny, we first consider binary perfect phylogenies: those perfect phylogenies for which the outdegree of any node, traversing from root to tips, is either 0 (leaves or taxa) or 2 (internal nodes). We then consider multifurcating perfect phylogenies in Sect. 4.

### Lattice structure of binary perfect phylogenies

The binary perfect phylogenies for a set of *n* tips possess a structure that will assist in enumerating binary ranked and unranked trees compatible with a set of sequences. In particular, we can make the set $\Pi _{n}$ of all binary perfect phylogenies of [*n*] into a **poset** by defining $\pi \le \sigma$ if either $\sigma$ is the same as $\pi$, or if $\sigma$ can be obtained by sequentially collapsing pairs of pendant edges, or cherries, of $\pi$. We then say $\pi$ is a **refinement** of $\sigma$. For example, $\pi =(2,3)$ refines $\sigma =(5)$. We say that two binary perfect phylogenies in $\Pi _{n}$ are **comparable** if they are equal or if one is a refinement of the other. An example of two perfect phylogenies that are not comparable is $\pi =(2,3)$ and $\sigma =(4,1)$.

Given two binary perfect phylogenies $\pi _{1}$ and $\pi _{2}$ in $\Pi _{n}$, their **meet**, denoted $\pi _{1} \wedge \pi _{2}$, is the largest perfect phylogeny that refines both $\pi _{1}$ and $\pi _{2}$. Similarly, the **join** of two binary perfect phylogenies $\pi _{1} \vee \pi _{2}$ is the smallest perfect phylogeny that is refined by both $\pi _{1}$ and $\pi _{2}$. Formal definitions of these notions appear in Definition 1.

**Fig. 4:**
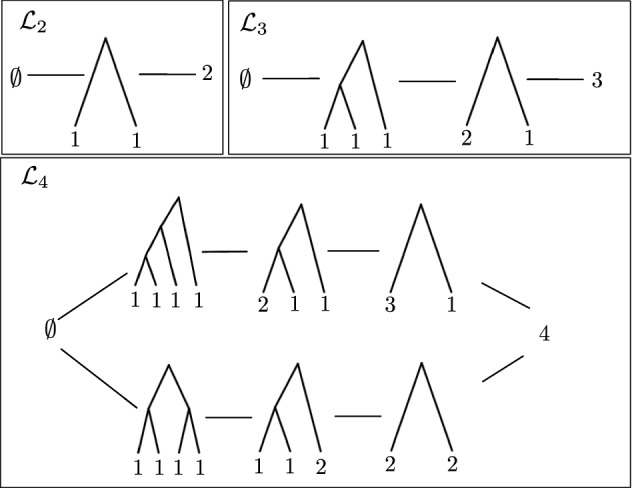
Hasse diagrams of the lattices of binary perfect phylogenies with $n=2$, 3, and 4 taxa

**Fig. 5:**
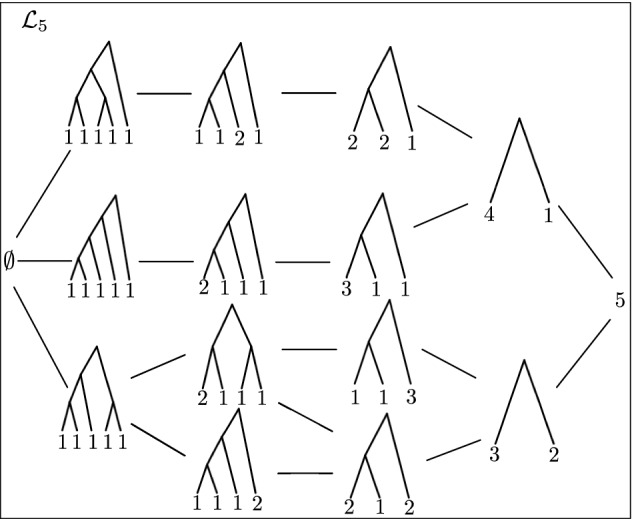
Hasse diagram of the lattice of binary perfect phylogenies with $n=5$ taxa

Under the meet and join operations, we will see in Theorem 5 that the poset $\Pi _{n} \cup \{\emptyset \}$ is a **lattice** ${\mathcal {L}}_n = (\Pi _{n} \cup \{\emptyset \}, \wedge , \vee )$. As a lattice, ${\mathcal {L}}_n$ possesses a **Hasse diagram** with a minimal and a maximal element. The **maximal** element of ${\mathcal {L}}_n$ is the single node perfect phylogeny (*n*) and the **minimal** element is $\emptyset$. Figures 4 and 5 show the Hasse diagrams of ${\mathcal {L}}_2$, ${\mathcal {L}}_3$, ${\mathcal {L}}_4$, ${\mathcal {L}}_5$.

#### Definition 1

(*Binary perfect phylogeny operations*). We define the binary perfect phylogeny symmetric operations $\wedge , \vee : (\cup _{n \ge 1} \Pi _{n} \cup \{ \emptyset \}) \times (\cup _{n \ge 1} \Pi _{n} \cup \{ \emptyset \}) \rightarrow (\cup _{n \ge 1} \Pi _{n} \cup \{ \emptyset \})$, where $\Pi _{n}$ is the space of binary perfect phylogenies of *n* leaves, as follows:

1. $$\pi \wedge \emptyset =\emptyset $$, for all $$\pi \in \Pi _{n}$$.
2. $$\pi \vee \emptyset = \pi $$, for all $$\pi \in \Pi _{n}$$.
3. $$\pi \wedge (n) = \pi $$, for all $$\pi \in \Pi _{n}$$.
4. $$\pi \vee (n) = (n)$$, for all $$\pi \in \Pi _{n}$$.
5. $\pi _1 \wedge \pi _2 = \emptyset$, for all $\pi _1 \in \Pi _{n_1}, \pi _2 \in \Pi _{n_2}$, with $n_1 \ne n_2$.
6. $\pi _1 \vee \pi _2 = \emptyset$, for all $\pi _1 \in \Pi _{n_1}, \pi _2 \in \Pi _{n_2}$, with $n_1 \ne n_2$.

The following proposition extends properties of the meet and join operations. It is proved in the “Appendix”.

#### Proposition 2

Let $\wedge , \vee$ be the two binary perfect phylogeny operations of Definition 1. Then:

1. Let $\pi _{1}=(n_{1},n_{2})$ and $\pi _{2}=(n_{3},n_{4})$ be two perfect phylogenies in $\Pi _{n}$ with $n_{1}+n_{2}=n_{3}+n_{4}=n$. Then
  $$\begin{aligned} \pi _{1} \vee \pi _{2}=(n_{1},n_{2}) \vee (n_{3},n_{4})={\left\{ \begin{array}{ll} (n_{1},n_{2}) &{} \text { if } n_{1}=n_{3} \text { or } n_{1}=n_{4}\\ (n) &{} \text { otherwise}. \\ \end{array}\right. } \end{aligned}$$
2. For all $\pi _{1}$, $\pi _{2}$, $\pi _{3}$, $\pi _{4}$ with $(\pi _{1},\pi _{2}) \in \Pi _{n}$ and $(\pi _{3},\pi _{4}) \in \Pi _{n}$,
  $$\begin{aligned} (\pi _{1},\pi _{2}) \wedge (\pi _{3},\pi _{4})= (\pi _{1}\wedge \pi _{3}, \pi _{2} \wedge \pi _{4}) \vee (\pi _{1}\wedge \pi _{4}, \pi _{2} \wedge \pi _{3}), \end{aligned}$$
  with the convention that $(\pi ,\emptyset )=\emptyset$. That is, the meet of two perfect phylogenies is the join of the two perfect phylogenies formed by merging two subtrees at the root. These four subtrees (two per newly formed perfect phylogeny) correspond to the meets of all pairs of subtrees, one from each of the original perfect phylogenies.
3. For all $\pi _{1}$, $\pi _{2}$, $\pi _{3}$, $\pi _{4}$ with $(\pi _{1},\pi _{2}) \in \Pi _{n}$ and $(\pi _{3},\pi _{4}) \in \Pi _{n}$, $\pi _{i}\in \Pi _{n_{i}}$ for $i=1,2,3,4$.
  $$\begin{aligned}&(\pi _{1},\pi _{2}) \vee (\pi _{3},\pi _{4})\\&\quad ={\left\{ \begin{array}{ll} (n) &{} \text { if } n_{1}\ne n_{3} \text { and } n_{1} \ne n_{4}\\ (\pi _{1}, \pi _{2} \vee \pi _{4}) &{} \text { if } \pi _{1}=\pi _{3}\\ (\pi _{1}, \pi _{2} \vee \pi _{3}) &{} \text { if } \pi _{1}=\pi _{4}\\ (\pi _{2}, \pi _{1} \vee \pi _{4}) &{} \text { if } \pi _{2}=\pi _{3}\\ (\pi _{2}, \pi _{1} \vee \pi _{3}) &{} \text { if } \pi _{2}=\pi _{4}\\ (\pi _{1}\vee \pi _{3}, \pi _{2} \vee \pi _{4}) \wedge (\pi _{1}\vee \pi _{4}, \pi _{2} \vee \pi _{3}) &{} \text { otherwise}, \end{array}\right. } \end{aligned}$$
  with the convention that $(\pi ,\emptyset )=\emptyset$. That is, the join of two perfect phylogenies is the meet of the two perfect phylogenies formed by merging two subtrees at the root. These four subtrees (two per newly formed perfect phylogeny) correspond to the joins of all pairs of subtrees, one from each of the original perfect phylogenies. In the particular case that the two original perfect phylogenies share one of the subtrees descending from the root, the join of the two perfect phylogenies is the perfect phylogeny that merges, at the root, the shared subtree with the join of the two different subtrees, one from each of the original perfect phylogenies. In the case that no two pairs of subtrees, one from each of the original perfect phylogenies, have the same size, the join is the maximal single node perfect phylogeny (*n*).
4. For all $$\pi _{1},\pi _{2}, \pi _{3} \in \Pi _{n},$$
  $$\begin{aligned} \pi _{1} \wedge (\pi _{2} \vee \pi _{3})=(\pi _{1} \wedge \pi _{2}) \vee (\pi _{1} \wedge \pi _{3}), \end{aligned}$$
  and
  $$\begin{aligned} \pi _{1} \vee (\pi _{2} \wedge \pi _{3})=(\pi _{1} \vee \pi _{2}) \wedge (\pi _{1} \vee \pi _{3}). \end{aligned}$$
5. Let $\pi , \sigma \in \Pi _{n}$ be two perfect phylogenies that are not comparable. There exist unique $\gamma ,\rho \in (\Pi _{n} \cup \{\emptyset \}) {\setminus } \{\pi ,\sigma \}$ such that
  $$\begin{aligned} \pi \wedge \sigma = \gamma , \quad \pi \vee \gamma = \pi , \quad \text { and }\quad \sigma \vee \gamma =\sigma , \end{aligned}$$
  and
  $$\begin{aligned} \pi \vee \sigma = \rho , \quad \pi \wedge \rho = \pi , \quad \text { and }\quad \sigma \wedge \rho =\sigma . \end{aligned}$$

Note that the meet and join operations are symmetric and that pairs $(\pi _1,\pi _2)$ are unordered; for convenience, we have expanded expressions in parts 1 and 3 of the proposition that could potentially be simplified using the symmetry.

We illustrate the operations in Definition 1 by considering a series of examples.

#### Example 3

**Fig. 6:**
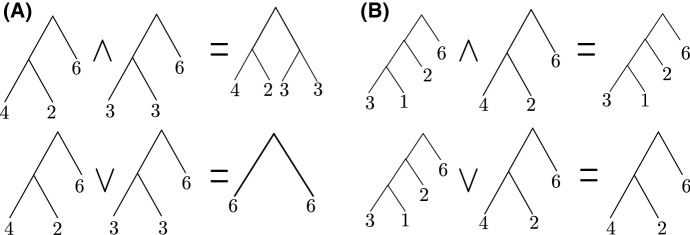
Examples of perfect phylogeny operations. **A** For perfect phylogenies ((4, 2), 6) and ((3, 3), 6), their meet is ((4, 2), (3, 3)), and their join is (6, 6). **B** For perfect phylogenies (((3, 1), 2), 6) and ((4, 2), 6), their meet is (((3, 1), 2), 6) and their join is ((4, 2), 6)

Consider $\pi _{1}=((4,2),6)$ and $\pi _{2}=((3,3),6)$ depicted in Fig. 6A. Their meet and join are given by:

$$\begin{aligned}&((4,2),6) \wedge ((3,3),6)\\&\quad = ((4,2)\wedge (3,3),6\wedge 6) \vee ((4,2)\wedge 6, 6 \wedge (3,3))\text { by Prop.~2 (2)} \\&\quad = (\emptyset ,6) \vee ((4,2),(3,3))\text { by Defn.~1 (3, 5) and Prop.~2 (2)}\\&\quad = \emptyset \vee ((4,2),(3,3)) \text { by convention}\\&\quad = ((4,2),(3,3)) \text { by Defn.~1 (2)}. \\&((4,2),6) \vee ((3,3),6)\\&\quad = (6, (4,2)\vee (3,3)) \text { by Prop.~2 (3)}\\&\quad = (6,6) \text { by Prop.~2 (1).} \end{aligned}$$

#### Example 4

For a more complex example, consider $\pi _{1}=((3,1),2),6)$ and $\pi _{2}=((4,2),6)$ depicted in Fig. 6B.

$$\begin{aligned}&(((3,1),2),6) \wedge ((4,2),6) \\&\quad = (((3,1),2)\wedge (4,2),6\wedge 6) \vee (((3,1),2)\wedge 6, 6 \wedge (4,2))\text { by Prop.~2 (2)}\\&\quad = (((3,1),2)\wedge (4,2),6) \vee (((3,1),2),(4,2)) \text { by Defn.~1 (3)} \\&\quad = ( ((3,1) \wedge 4, 2\wedge 2),6) \vee (((3,1),2),(4,2)) \text { by Defn.~1 (2, 5) and Prop.~2 (2)} \\&\quad = (((3,1),2),6) \vee (((3,1),2),(4,2)) \text { by Defn.~1 (3)} \\&\quad = (((3,1),2),6) \text { by Defn.~1 (4) and Prop.~2 (3)}. \\&((3,1),2),6) \vee ((4,2),6)\\&\quad = (((3,1),2)\vee (4,2),6) \text { by Prop.~2 (3)} \\&\quad = ((4,2),6) \text { by Defn.~1 (4) and Prop.~2 (3)}. \end{aligned}$$

To make use of the operations $\wedge$ and $\vee$ for counting binary ranked and unranked trees compatible with a perfect phylogeny, we need a theorem that shows that the two operations $\wedge$ and $\vee$ induce the same order. That is, we will show that $(\Pi _{n} \cup \{\emptyset \}, \wedge ,\vee )$ is a lattice.

A *lattice* (Nation 1998) is an algebra ${\mathcal {L}}(L,\wedge ,\vee )$ satisfying, for all $x,y,z \in L$,

1. $$x \wedge x =x$$ and $$x \vee x=x$$,
2. $$x \wedge y =y \wedge x$$ and $$x \vee y=y \vee x$$,
3. $$x \wedge (y \wedge z) = (x \wedge y) \wedge z$$ and $$x \vee (y \vee z)=(x\vee y) \vee z$$,
4. $$x \wedge (x \vee y)=x$$ and $$ x\vee (x \wedge y)=x$$.

In the “Appendix,” we verify these conditions for $(\Pi _{n} \cup \{\emptyset \},\wedge ,\vee )$, giving the following theorem.

#### Theorem 5

$$(\Pi _{n} \cup \{\emptyset \},\wedge ,\vee )$$ is a lattice.

### Unranked unlabeled tree shapes compatible with a binary perfect phylogeny

With the lattice structure of the binary perfect phylogenies established, we are now equipped to calculate the number of compatible unranked unlabeled tree shapes with *n* leaves. Notice that an unranked unlabeled tree shape can be transformed into a perfect phylogeny with the same number of tips by assigning the count 1 to all leaves. We use ${\mathcal {P}}(T_{n})$ to denote the perfect phylogeny with *n* tips that corresponds to the unranked unlabeled tree shape $T_{n}$.

#### Definition 6

(*Unranked unlabeled tree shape* $T_{n}$ *compatible with a perfect phylogeny* $\pi \in \Pi _{n}$). An unranked unlabeled tree shape with *n* leaves, $T_{n}$, is compatible with a perfect phylogeny $\pi \in \Pi _{n}$, if (1) a one-to-one correspondence exists between the *k* leaves of $\pi$ with leaf counts $n_{1},n_{2},\ldots ,n_{k}$ and *k* disjoint subtrees of $T_{n}$ containing $n_{1},n_{2},\ldots ,n_{k}$ leaves, respectively; and (2) ${\mathcal {P}}(T_{n})\le \pi$, that is, ${\mathcal {P}}(T_{n})$ is a refinement of $\pi$.

**Fig. 7:**
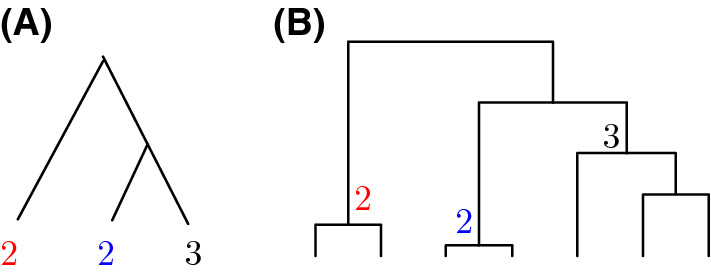
Example of a tree shape compatible with a perfect phylogeny. **A** A perfect phylogeny. **B** An unranked unlabeled tree shape that is compatible with the perfect phylogeny in (**A**). The numbers indicate the one-to-one correspondence described in Definition 6

We use the symbol ${\mathcal {G}}_{c}(\pi )=\{T_{n}:T_{n} \rightsquigarrow \pi \}$ to denote the set of unranked unlabeled tree shapes compatible with a perfect phylogeny $\pi \in \Pi _{n}$. For a perfect phylogeny $\pi$ consisting of a single leaf with leaf count *n*, the number of compatible unranked unlabeled tree shapes is simply the number of unranked unlabeled tree shapes of size *n*, or $|{\mathcal {G}}_{c}(\pi )| = S_n$. Figure 7 shows an example of an unranked unlabeled tree shape compatible with a perfect phylogeny of sample size 7.

#### Proposition 7

For $n_1,n_2 \ge 1$, the number of unranked unlabeled tree shapes compatible with a cherry perfect phylogeny $(n_{1},n_{2}) \in \Pi _{n}$ is

6 $$\begin{aligned} |{\mathcal {G}}_{c}((n_{1},n_{2}))|={\left\{ \begin{array}{ll} S_{n_{1}}S_{n_{2}} &{}\quad \text {if } n_{1} \ne n_{2}\\ \frac{1}{2}S_{n_{1}}(S_{n_{1}}+1) &{}\quad \text {if } n_{1} = n_{2}. \end{array}\right. } \end{aligned}$$

#### Proof

By Definition 6, an unranked unlabeled tree shape is compatible with the perfect phylogeny $\pi = (n_1,n_2)$ if it possesses two subtrees, one with $n_1$ leaf descendants and another with $n_2$ leaf descendants. Decomposing an unranked unlabeled tree shape at its root, the number of shapes with this property is $S_{n_{1}}S_{n_{2}}$ for $n_1 \ne n_2$ and $\frac{1}{2}S_{n_{1}}(S_{n_{1}}+1)$ for $n_1=n_2$. $\square$

#### Proposition 8

For $n_1,n_2 \ge 1$ and $\pi _{1} \in \Pi _{n_{1}}$, $\pi _{2} \in \Pi _{n_{2}}$, the number of unranked unlabeled tree shapes compatible with a binary perfect phylogeny $\pi =(\pi _{1},\pi _{2}) \in \Pi _{n}$ is

7 $$\begin{aligned}&|{\mathcal {G}}_{c}((\pi _{1},\pi _{2}))|\nonumber \\&\quad = {\left\{ \begin{array}{ll} |{\mathcal {G}}_{c}(\pi _{1})| \, |{\mathcal {G}}_{c}(\pi _{2})|-\frac{1}{2}|{\mathcal {G}}_{c}(\pi _{1} \wedge \pi _{2})| \, (|{\mathcal {G}}_{c}(\pi _{1} \wedge \pi _{2})|-1) &{} \text {if } \pi _{1} \wedge \pi _{2} \ne \emptyset \\ |{\mathcal {G}}_{c}(\pi _{1})| \, |{\mathcal {G}}_{c}(\pi _{2})| &{} \text {if } \pi _{1} \wedge \pi _{2}= \emptyset . \\ \end{array}\right. }\qquad \end{aligned}$$

#### Proof

If $\pi _{1} \wedge \pi _{2}=\emptyset$, then no tree shapes are compatible with both $\pi _{1}$ and $\pi _{2}$. Hence, the number of tree shapes compatible with $(\pi _{1},\pi _{2})$ is simply the product of the number of tree shapes compatible with $\pi _{1}$ and the number of tree shapes compatible with $\pi _{2}$.

If $\pi _{1} \wedge \pi _{2} \ne \emptyset$, then certain tree shapes can be compatible with both $\pi _{1}$ and $\pi _{2}$, i.e., compatible with $\pi _{1} \wedge \pi _{2}$. We sum four quantities. (1) Consider the set of tree shapes compatible with both perfect phylogenies $\pi _{1}$ and $\pi _{2}$. They can either be assigned the same tree shape, in $|{\mathcal {G}}_{c}(\pi _{1} \wedge \pi _{2})|$ ways, or they can be assigned different tree shapes, in $\frac{1}{2}(|{\mathcal {G}}_{c}(\pi _{1} \wedge \pi _{2})|^{2}-|{\mathcal {G}}_{c}(\pi _{1} \wedge \pi _{2})|)$ ways, resulting in $\frac{1}{2}|{\mathcal {G}}_{c}(\pi _{1} \wedge \pi _{2})|(|{\mathcal {G}}_{c}(\pi _{1} \wedge \pi _{2})|+1)$ tree shapes. (2) If $\pi _{2}$ is a refinement of $\pi _{1}$ and $\pi _{1} \ne \pi _{2}$, then there are $|{\mathcal {G}}_{c}(\pi _{1} \wedge \pi _{2})| (|{\mathcal {G}}_{c}(\pi _{1})|-|{\mathcal {G}}_{c}(\pi _{1} \wedge \pi _{2})|)$ tree shapes. (3) Similarly, if $\pi _{1}$ is a refinement of $\pi _{2}$ and $\pi _{1} \ne \pi _{2}$, then there are $|{\mathcal {G}}_{c}(\pi _{1} \wedge \pi _{2})| (|{\mathcal {G}}_{c}(\pi _{2})|-|{\mathcal {G}}_{c}(\pi _{1} \wedge \pi _{2})|)$. (4) If $\pi _{1}$ and $\pi _{2}$ are not comparable, that is, if neither is a refinement of the other, then there are $(|{\mathcal {G}}_{c}(\pi _{1})|- |{\mathcal {G}}_{c}(\pi _{1} \wedge \pi _{2})|) (|{\mathcal {G}}_{c}(\pi _{2})|-|{\mathcal {G}}_{c}(\pi _{1} \wedge \pi _{2})|)$ tree shapes. Scenarios (2), (3), and (4) are mutually exclusive, and only one of the quantities in (2), (3), and (4) is nonzero; summing the four quantities gives the result. $\square$

Propositions 7 and 8 provide a recursive formula for calculating the number of tree shapes compatible with a binary perfect phylogeny. For example, examining Fig. 6A, the number of tree shapes compatible with (4, 2) is $S_{4}S_{2}=2$, and the number of tree shapes compatible with ((4, 2), 6) is $|{\mathcal {G}}_{c}(4,2)| \, |{\mathcal {G}}_{c}(6)|- \frac{1}{2}|{\mathcal {G}}_{c}(4,2)| \, (|{\mathcal {G}}_{c}(4,2)|-1) =(2) (6)-\frac{1}{2}(2)(1)=11.$ Table 1 shows the number of tree shapes compatible with certain perfect phylogenies of sample size 10.

### Ranked unlabeled tree shapes compatible with a binary perfect phylogeny

Next, for a binary perfect phylogeny, we compute the number of compatible ranked unlabeled tree shapes with *n* leaves.

#### Definition 9

(*Ranked unlabeled tree shape* $T^{R}_{n}$ *compatible with a perfect phylogeny* $\pi \in \Pi _{n}$). A ranked unlabeled tree shape with *n* leaves, $T^{R}_{n}$, is compatible with a perfect phylogeny $\pi \in \Pi _{n}$ if the unranked unlabeled tree shape $T_{n}$ obtained by removing the ranking from ${T}^{R}_{n}$ is compatible with $\pi$.

#### Proposition 10

For $n_1, n_2 \ge 1$, the number of ranked unlabeled tree shapes compatible with a cherry perfect phylogeny $(n_{1},n_{2}) \in \Pi _{n}$ is

8 $$\begin{aligned} \big |{\mathcal {G}}^{T}_{c}((n_{1},n_{2}))\big |{=}{\left\{ \begin{array}{ll} \left( {\begin{array}{c}n_{1}+n_{2}-2\\ n_{1}-1\end{array}}\right) R_{n_{1}}R_{n_{2}} &{}\quad \text {if } n_{1} \ne n_{2}\\ \frac{1}{2}\left( {\begin{array}{c}2n_{1}-2\\ n_{1}-1\end{array}}\right) R^{2}_{n_{1}}&\quad \text {if } n_{1} = n_{2}. \end{array}\right. } \end{aligned}$$

#### Proof

By Definition 9, a ranked unlabeled tree shape $T^R$ is compatible with the perfect phylogeny $\pi = (n_1,n_2)$ if the associated unranked unlabeled tree shape *T* obtained by removing the ranking of $T^R$ is compatible with $\pi$. By Definition 6, the unranked unlabeled tree shape *T* is compatible with the perfect phylogeny $\pi = (n_1,n_2)$ if it possesses two subtrees, one with $n_1$ leaf descendants and another with $n_2$ leaf descendants.

We decompose a ranked unlabeled tree at its root into subtrees of size $n_1$ and $n_2$. If $n_{1} \ne n_{2}$, then the $n_{1}-1$ interior nodes of the subtree with $n_{1}$ leaves and the $n_{2}-1$ interior nodes of the subtree with $n_{2}$ leaves can be interleaved in $$\left( {\begin{array}{c}n_{1}+n_{2}-2\\ n_{1}-1\end{array}}\right) $$ ways. If $n_{1}=n_{2}$, then the two ranked subtrees can be the same in $R_{n_{1}}$ ways, each with $$\frac{1}{2}\left( {\begin{array}{c}2n_{1}-2\\ n_{1}-1\end{array}}\right) $$ ways of interleaving the two ranked unlabeled subtrees; the two ranked subtrees can differ in $\frac{1}{2}(R^{2}_{n_{1}}-R_{n_{1}})$ ways, each with $$\left( {\begin{array}{c}2n_{1}-2\\ n_{1}-1\end{array}}\right) $$ ways of interleaving the subtrees. $\square$

#### Proposition 11

For $n_1, n_2 \ge 1$ and $\pi _1 \in \Pi _{n_1}, \pi _2 \in \Pi _{n_2}$, the number of ranked unlabeled tree shapes compatible with a binary perfect phylogeny $\pi =(\pi _{1},\pi _{2}) \in \Pi _{n}$ is

9 $$\begin{aligned} \big |{\mathcal {G}}^{T}_{c}((\pi _1, \pi _2))\big |={\left\{ \begin{array}{ll} \left( {\begin{array}{c}2 n_{1}-2\\ n_{1}-1\end{array}}\right) \big (\big |{\mathcal {G}}^{T}_{c}(\pi _{1})\big | \, \big |{\mathcal {G}}^{T}_{c}(\pi _{2})\big | -\frac{1}{2}\big |{\mathcal {G}}^{T}_{c}(\pi _{1} \wedge \pi _{2})\big |^{2}\big ) &{} \text { if } \pi _{1} \wedge \pi _{2} \ne \emptyset \\ \left( {\begin{array}{c}n_{1}+n_{2}-2\\ n_{1}-1\end{array}}\right) \big |{\mathcal {G}}^{T}_{c}(\pi _{1})\big | \, \big |{\mathcal {G}}^{T}_{c}(\pi _{2})\big |&\text { if } \pi _{1} \wedge \pi _{2}= \emptyset . \end{array}\right. } \end{aligned}$$

#### Proof

If $\pi _{1} \wedge \pi _{2} = \emptyset$, then the number of ranked tree shapes compatible with $(\pi _{1},\pi _{2})$ is simply the product of the number of ranked tree shapes compatible with $\pi _{1}$, the number of ranked tree shapes compatible with $\pi _{2}$, and the number of ways of interleaving their rankings.

If $\pi _{1} \wedge \pi _{2} \ne \emptyset$, then certain ranked tree shapes can be compatible with both $\pi _{1}$ and $\pi _{2}$, i.e., compatible with $\pi _{1}\wedge \pi _{2}$. We therefore have three cases: the two perfect phylogenies are the same, one is a refinement of the other (two possible ways), or neither is a refinement of the other. The cardinalities in these cases are $\frac{1}{2}|{\mathcal {G}}^{T}_{c}(\pi _{1} \wedge \pi _{2})|^{2}$, $|{\mathcal {G}}^{T}_{c}(\pi _{1} \wedge \pi _{2})| \, (|{\mathcal {G}}^{T}_{c}(\pi _{2})|-|{\mathcal {G}}^{T}_{c}(\pi _{1} \wedge \pi _{2})|)+|{\mathcal {G}}^{T}_{c}(\pi _{1} \wedge \pi _{2})|(|{\mathcal {G}}^{T}_{c}(\pi _{1})|-|{\mathcal {G}}^{T}_{c}(\pi _{1} \wedge \pi _{2})|)$, and $(|{\mathcal {G}}^{T}_{c}(\pi _{1})|-|{\mathcal {G}}^{T}_{c}(\pi _{1} \wedge \pi _{2})|)(|{\mathcal {G}}^{T}_{c}(\pi _{2})|-|{\mathcal {G}}^{T}_{c}(\pi _{1} \wedge \pi _{2})|)$, respectively, all multiplied by the possible number of interleavings of the rankings $$\left( {\begin{array}{c}2n_{1}-2\\ n_{1}-1\end{array}}\right) $$. $\square$

Propositions 10 and 11 provide a recursive formula for calculating the number of ranked tree shapes compatible with a binary perfect phylogeny. For Fig. 6A, the number of ranked tree shapes compatible with (4, 2) is $(4)(2)=8$, and the number of ranked tree shapes compatible with ((4, 2), 6) is $$\left( {\begin{array}{c}10\\ 5\end{array}}\right) (|{\mathcal {G}}^{T}_{c}(4,2)| \, |{\mathcal {G}}^{T}_{c}(6)|- \frac{1}{2}|{\mathcal {G}}^{T}_{c}(4,2)|^2) =\left( {\begin{array}{c}10\\ 5\end{array}}\right) [(8)(16)-\frac{1}{2}(8)^{2}]=24,\!\!192$$.

Table 1 shows the number of ranked unlabeled tree shapes compatible with some of the perfect phylogenies of sample size 10. We can observe that these numbers exceed corresponding numbers of unranked unlabeled tree shapes compatible with the perfect phylogenies, just as the numbers of ranked unlabeled tree shapes exceed the numbers of unranked unlabeled tree shapes (Sect. 2.4).

For the ranked unlabeled tree shapes compatible with a binary perfect phylogeny, we can examine the asymptotic growth of the number of compatible ranked unlabeled tree shapes in particular families of binary perfect phylogenies. For a fixed integer value $x \ge 1$, consider the family of binary perfect phylogenies $B_x(n)=(x,n-x)$ as *n* increases. These are cherry phylogenies with labels *x* and $n-x$ at their two leaves. Let $b_x(n)$ be the number of ranked unlabeled tree shapes compatible with $B_x(n)$. Among the integer sequences $b_1(n)$, $b_2(n)$, $b_3(n)$, $\ldots$, the next proposition shows that $b_2(n)$ has the fastest asymptotic growth. In other words, as *n* grows large, the value of *x* for which the number of ranked unlabeled tree shapes compatible with the perfect phylogeny $B_x(n)$ is asymptotically largest is $x=2$.

#### Proposition 12

Among the integer sequences $b_1(n)$, $b_2(n)$, $b_3(n)$, $\ldots$, the sequence $b_2(n)$ has the fastest asymptotic growth.

#### Proof

For a fixed integer value $x \ge 0$, let $\beta _x = (x+1,n-x+1)$ be a binary perfect phylogeny with two leaves, labeled by $x+1$ (say to the left of the root) and $n-x+1$ (to the right of the root). The set of ranked unlabeled tree shapes compatible with $\beta _x$ corresponds to the set of ranked unlabeled tree shapes with $n+1$ internal nodes ($n+2$ leaves), *x* internal nodes for the left root subtree, and $n-x$ internal nodes for the right root subtree.

We consider an increasing sequence of values of *n*. Supposing $n > 2x$ so that the root subtrees of $\beta _x$ cannot have the same sample size, we apply Proposition 11, finding that the number of ranked unlabeled tree shapes compatible with $\beta _x$ is

10 $$\begin{aligned} {{n}\atopwithdelims (){x}} e_x e_{n-x}, \end{aligned}$$

where $e_i$ is the number of ranked unlabeled tree shapes with *i* internal nodes. Following Eq. 3, the integer $e_i$ is the *i*th Euler number, $e_i=R_{i+1}$.

The exponential generating function of the sequence $(e_i)$ is (Brent and Harvey 2013)

11 $$\begin{aligned} \sum _{i=0}^{\infty } \frac{e_i z^i}{i!} = \sec (z) + \tan (z). \end{aligned}$$

We can write the ratio $q_i=\frac{e_i}{i!}$ as (Flajolet and Sedgewick 2009, p. 269; Brent and Harvey 2013)

12 $$\begin{aligned} q_i = \left\{ \begin{array}{l l} 2 \left( \frac{2}{\pi } \right) ^{i+1} \sum \limits _{k=0}^{\infty }\frac{(-1)^{k}}{(2k+1)^{i+1}} , &{}\quad \text {if } i \text { is even} \\ 2 \left[ \left( \frac{2}{\pi } \right) ^{i+1} - \left( \frac{1}{\pi } \right) ^{i+1} \right] \sum \limits _{k=1}^{\infty } \frac{1}{k^{i+1}} , &{}\quad \text {if } i \text { is odd}. \\ \end{array} \right. \end{aligned}$$

As *i* becomes large, by applying singularity analysis to Eq. 11, or by computing directly from Eq. 12, we have the asymptotic relation

13 $$\begin{aligned} q_i \sim 2 \left( \frac{2}{\pi } \right) ^{i+1}. \end{aligned}$$

With $q_x = e_x/x!$, we rewrite Eq. 10 as $n! \, q_x q_{n-x}$. Letting $n \rightarrow \infty$ for a fixed *x*, we can use Eq. 12 to rewrite $q_x$, and because *x* is constant as *n* grows, we can use Eq. 13 for the asymptotic value of $q_{n-x}$. Hence, for increasing values of *n*, the number of ranked tree shapes compatible with the perfect phylogeny $\beta _x$ behaves asymptotically like the product of *n*! and

14 $$\begin{aligned} q_x q_{n-x} \sim 4 \left( \frac{2}{\pi } \right) ^{n+2} c_x, \end{aligned}$$

where

15 $$\begin{aligned} c_x = \left\{ \begin{array}{ll} \sum \limits _{k=0}^{\infty }\frac{(-1)^{k}}{(2k+1)^{x+1}} , &{}\quad \text {if } x \text { is even} \\ \left( 1 - \frac{1}{2^{x+1}} \right) \sum \limits _{k=1}^{\infty } \frac{1}{k^{x+1}} , &{}\quad \text {if } x \text { is odd}. \\ \end{array} \right. \end{aligned}$$

Note that $\zeta (s) = \sum _{k=1}^{\infty } \frac{1}{k^s}$ is the Riemann zeta function. If *x* is even, then

16 $$\begin{aligned} c_x = 1 + \left( - \frac{1}{3^{x+1}} + \frac{1}{5^{x+1}} \right) + \left( - \frac{1}{7^{x+1}} + \frac{1}{9^{x+1}} \right) + ... \le 1. \end{aligned}$$

Among odd values of *x*, we have $c_1= \frac{3}{4} \, \zeta (2) = \pi ^2/8 \approx 1.2337$ for $x=1$. For odd $x\ge 3$, we have

$$\begin{aligned} c_x< \zeta (x+1) \le \zeta (3) \approx 1.2021 < c_1. \end{aligned}$$

Hence, $c_1 > 1$ exceeds $c_x$ both for even *x* and for all odd $x \ge 3$.

Because $c_x$ has its maximum at $x=1$, from Eq. 14, we conclude that the product $q_x q_{n-x}$ grows asymptotically fastest for $x=1$. In particular, as $n \rightarrow \infty$, the value of *x* for which the binary perfect phylogeny $\beta _x$ has the largest number of compatible ranked unlabeled tree shapes is $x=1$—that is, when $\beta _x = \beta _1=(2,n)$. $\square$

In Table 1, we can observe an example of Proposition 12. The value of $b_2(10)$, or 2176, exceeds the values of $b_x(10)$ for all other values of *x* (with the trivial exception that $b_2(10)=b_8(10)$). The asymptotic approximation from Eq. 14 gives

17 $$\begin{aligned} b_2(n) \sim 2 \bigg (\frac{2}{\pi }\bigg )^{n-2} (n-2)!, \end{aligned}$$

which, for $n=10$, yields $b_2(10) \approx 20,643,840/\pi ^8 \approx 2175.66$.

We also obtain the following corollary.

#### Corollary 13

Among the integer sequences $b_1(n)$, $b_2(n)$, $b_3(n)$, $\ldots$, the sequence $b_1(n)$ has the slowest asymptotic growth.

#### Proof

From the proof of Proposition 12, for $x \ge 0$, $b_{x+1}(n+2)$ gives the number of ranked unlabeled shapes compatible with $\beta _x=(x+1,n-x+1)$. The proof obtains $b_{x+1}(n+2) = n! \, q_x q_{n-x}$, or, following Eq. 14, $b_{x+1}(n+2) \sim 4 (\frac{2}{\pi })^{n+2} c_x n!$, where $c_x$ follows Eq. 15. Hence, to show $b_1(n)$ has the slowest growth among $b_1(n)$, $b_2(n)$, $b_3(n)$, $\ldots$, it suffices to show that in Eq. 15, among all values of $x \ge 0$, $c_0$ is the smallest.

We see that $c_0$ is equal to the power series expansion of $\arctan (1)$, or $\pi /4$. For even $x \ge 2$, consider the expansion of $c_x$ in Eq. 16, and let $f(x,A)=-{1}/{A^{x+1}}+{1}/{(A+2)^{x+1}}$, so that $c_x = 1 + f(x,3) + f(x,7) + f(x,11) + \ldots$. We claim that termwise, for even $x \ge 2$ and $A \ge 3$, $f(x,A) > f(0,A)$, so that summing terms in Eq. 16, we obtain $c_x > c_0$ for even $x \ge 2$.

To prove the claim, it suffices to show that for fixed $A \ge 3$, *f* increases from $x=0$, or $\partial f(x,A) / \partial x > 0$ for $x > 0$. We have $\partial f(x,A) / \partial x = (\log A)/A^{x+1} - [\log (A+2)]/(A+2)^{x+1}$. To verify that $\partial f(x,A) / \partial x > 0$ for $x > 0$ and $A \ge 3$, we see that $\partial f(x,A) / \partial x > 0$ is equivalent to $(\frac{A+2}{A})^{x+1} > \frac{\log (A+2)}{\log A}$. Now, from the inequality $1+y \le e^y$, we obtain $1+\frac{2}{A} \le e^{2/A}$ and hence $(\frac{A+2}{A})^A \le e^2$. Because $A \ge 3$, $A^2 > e^2$. Therefore, $A^2 > (\frac{A+2}{A})^A$, from which $A^{A+2} > (A+2)^A$, $(A+2)\log A > A \log (A+2)$, and $\frac{A+2}{A} > \frac{\log (A+2)}{\log A}$. We then have $(\frac{A+2}{A})^{x+1}> \frac{A+2}{A} > \frac{\log (A+2)}{\log A}$, and $\partial f(x,A) / \partial x > 0$.

We conclude $c_x > c_0$ for all even $x \ge 2$. From the proof of Proposition 12, we know $c_1 = {\pi ^2}/{8} > \pi /4 = c_0$. For odd $x \ge 3$, in Eq. 15, $\sum _{k=1}^\infty {1}/{k^{x+1}} > 1$, so that $c_x> 1 - {1}/{2^{x+1}} \ge 1 - {1}/{2^{3+1}} = \frac{15}{16} > {\pi }/{4} = c_0$, completing the proof. $\square$

The proof of Corollary 13 yields

18 $$\begin{aligned} b_1(n) \sim 2 \bigg (\frac{2}{\pi }\bigg )^{n-1} (n-2)!. \end{aligned}$$

The approximation yields $b_1(10) \approx 41{,}287{,}680/\pi ^9 \approx 1385.07$, and we see in Table 1 that $b_1(10)=1385$. From Eqs. 17 and 18, we have $b_2(n)/b_1(n) \sim \pi /2$, and we see from Table 1 that $b_2(10)/b_1(10) = 2176/1385 \approx 1.57 \approx \pi /2$.

### Ranked labeled tree shapes compatible with a labeled binary perfect phylogeny

Propositions 7, 8, 10 and 11 provide recursive formulas for enumerating unranked unlabeled tree shapes and ranked unlabeled tree shapes compatible with a binary perfect phylogeny. In these cases, a perfect phylogeny representation does not use individual sequence labels; the labels of the tips of the perfect phylogeny are simply counts of numbers of sequences. We now consider **labeled perfect phylogenies** that partition the set of labeled individual sequences. We still use the parenthetical notation described in Sect. 2.3 to denote a labeled perfect phylogeny, for example $\pi =(2,3)$, however, it must be understood that this labeled perfect phylogeny partitions the sampled sequences into two different sets of labeled sequences.

**Fig. 8:**
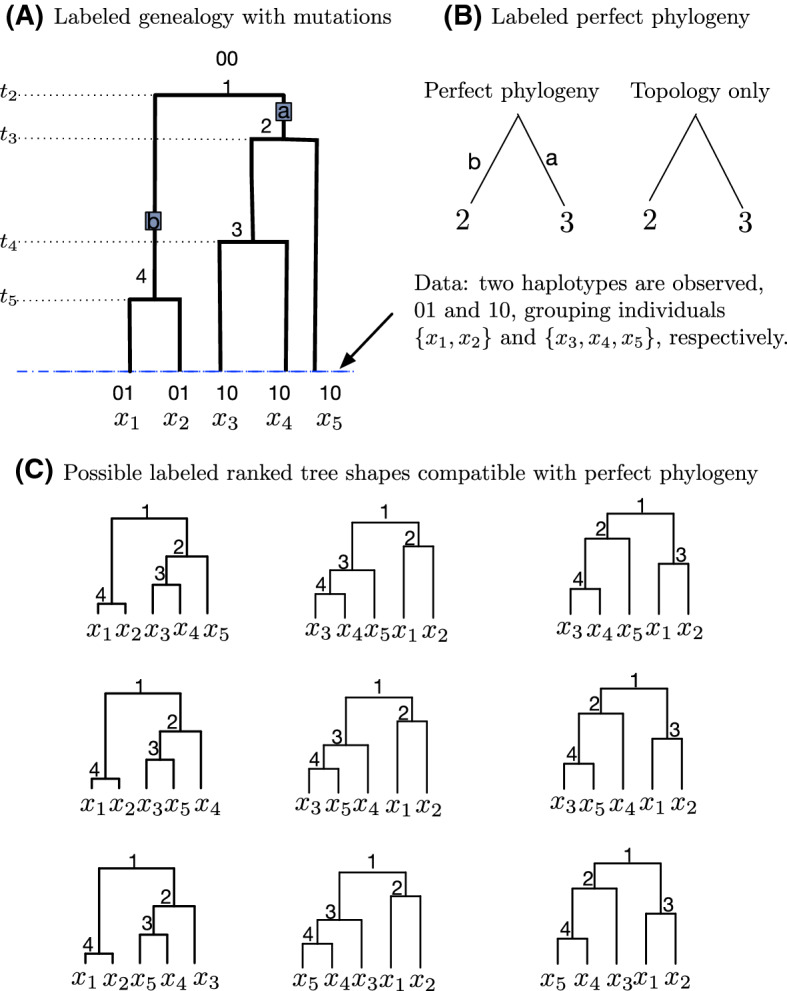
Coalescent and infinitely-many-sites generative model of binary molecular data. **A** A genealogy of 5 individuals, with 2 superimposed mutations depicted as gray squares. The root is labeled by the ancestral type 00, and the leaves are labeled by the genetic type at each of three mutated sites. The first two leaves from left to right are labeled 01 because one mutation occurs in their path to the root. The third, fourth and fifth individuals have one mutation in their path to the root and are labeled 10. The order and label of the mutations is unimportant; however, individual labels $x_{1},x_{2},x_{3},x_{4},x_{5}$ are important. For ease of exposition, we label the mutations a, b. The first site corresponds to mutation a, and the second to b. **B** Left, a labeled perfect phylogeny representation of the observed data at the tips of (**A**). Data consist of 2 unique haplotypes 01 and 10, with frequencies 2 and 3, respectively. The corresponding frequencies are the labels of tips of the perfect phylogeny; however, it is understood that the two leaves correspond to $\{x_{1},x_{2}\}$ and $\{x_{3},x_{4},x_{5}\}$ respectively. Right, perfect phylogeny topology obtained by removing the edge labels of the perfect phylogeny. **C** The nine ranked labeled tree shapes compatible with the labeled perfect phylogeny topology in (**B**). Note that in (**C**), if we ignore the branching order and drop the internal node labels, in each row, the three trees are equivalent—so that each row corresponds to one of the three *unranked* labeled tree shapes compatible with the labeled perfect phylogeny topology in (**B**)

Consider $\{x_{1},x_{2}\}$ and $\{x_{3},x_{4},x_{5}\}$ in the perfect phylogeny of Fig. 8B. We are now interested in calculating the number of ranked labeled tree shapes compatible with a labeled binary perfect phylogeny. Figure 8C shows all the ranked labeled tree shapes compatible with the labeled perfect phylogeny. For ranked labeled tree shapes, the enumeration follows a simple recursive expression.

#### Definition 14

(*Ranked labeled tree shape* $T^{L}_{n}$ *compatible with a labeled perfect phylogeny* $\pi \in \Pi ^{L}_{n}$). A ranked labeled tree shape with *n* leaves, $T^{L}_{n}$, is compatible with a perfect phylogeny $\pi \in \Pi ^{L}_n$ if the unranked unlabeled tree shape $T_{n}$ obtained by removing the ranks and the labels from ${T}^{L}_{n}$ is compatible with $\pi$ and the one-to-one correspondence between the *k* leaves of $\pi$ and the *k* disjoint subtrees of $T^{L}_{n}$ correspond to the same partition of the individual sequences.

#### Proposition 15

For $n_1, n_2 \ge 1$ and $\pi _{1}\in \Pi _{n_{1}}^L, \pi _{2}\in \Pi _{n_{2}}^L$ the number of ranked labeled tree shapes compatible with a labeled binary perfect phylogeny $\pi =(\pi _{1},\pi _{2})$ is

19 $$\begin{aligned} |{\mathcal {G}}^{L}_{c}(\pi )|= \left( {\begin{array}{c}n_{1}+n_{2}-2\\ n_{1}-1\end{array}}\right) \big |{\mathcal {G}}^{L}_{c}(\pi _{1})\big | \, \big |{\mathcal {G}}^{L}_{c}(\pi _{2})\big |. \end{aligned}$$

#### Proof

We can count the number of ranked labeled tree shapes by dividing $\pi$ at the root into two subtrees, one with $n_1$ leaves and perfect phylogeny $\pi _1$, and the other with $n_2$ leaves and perfect phylogeny $\pi _2$, both partitioning the sampled sequences. The number of such trees is the product of the numbers of ranked labeled trees for the two subtrees and the number of ways of interleaving the internal nodes of the two subtrees. In this case, the two perfect phylogenies $\pi _{1}$ and $\pi _{2}$ can never be identical because they correspond to different sets of sequences. $\square$

Counts for the number of ranked labeled tree shapes for some of the perfect phylogenies of 10 taxa (with an arbitrary labeling) appear in Table 1. Given a perfect phylogeny in the table, we can observe that the number of ranked labeled tree shapes far exceeds the number of ranked unlabeled tree shapes.

Continuing with ((4,2),6), the number of ranked labeled tree shapes compatible with this (arbitrarily labeled) perfect phylogeny is ${10 \atopwithdelims ()5} |{\mathcal {G}}_c^L((4,2))| \, |{\mathcal {G}}_c^L((6))| = {10 \atopwithdelims ()5} {4 \atopwithdelims ()3} \, |{\mathcal {G}}_c^L((4))|\, |{\mathcal {G}}_c^L((2))| \,|{\mathcal {G}}_c^L((6))| = {10 \atopwithdelims ()5} {4 \atopwithdelims ()3} L_4 L_2 L_6 = 252 \times 4 \times 18 \times 1 \times 2700= 48{,}988{,}800$.

We can obtain a result analogous to Proposition 12; we characterize, for binary labeled perfect phylogenies $B_x(n)=(x,n-x)$, the one compatible with the largest number of ranked labeled tree shapes. Let $b_x'(n)$ denote the number of ranked labeled tree shapes compatible with $B_x(n)$.

#### Proposition 16

Fix $n \ge 2$. Among the values $b_1'(n), b_2'(n), \ldots , b_{\lfloor \frac{n}{2} \rfloor }'(n)$, the largest is $b_1'(n)$, and the smallest is $b_{\lfloor \frac{n}{2} \rfloor }'(n)$.

#### Proof

Applying Proposition 15, we have $b_x'(n)={n-2 \atopwithdelims ()x-1} \, L_x \, L_{n-x}$. Simplifying with Eq. 1, we obtain $b_x'(n) = [n! \, (n-2)! / {2^{n-2}}]{n \atopwithdelims ()x}^{-1}$. As it is quickly verified that the binomial coefficients ${n \atopwithdelims ()x}$ increase monotonically from $x=1$ to $x=\lfloor \frac{n}{2} \rfloor$, $b_x'$ decreases monotonically from $x=1$ to $x=\lfloor \frac{n}{2} \rfloor$. $\square$

An example of Proposition 16 is visible in Table 1, in which $b_1'(10)=57{,}153{,}600$ exceeds $b_2'(10)$, $b_3'(10)$, $b_4'(10)$, and $b_5'(10)$, among which $b_5'(10)=2{,}268{,}000$ is the smallest.

### Unranked labeled tree shapes compatible with a labeled binary perfect phylogeny

Continuing with the labeled perfect phylogenies from Sect. 3.4, we now count the unranked labeled binary perfect phylogenies compatible with a labeled binary perfect phylogeny.

Consider $\{x_{1},x_{2}\}$ and $\{x_{3},x_{4},x_{5}\}$ in the perfect phylogeny of Fig. 8B. We calculate the number of unranked labeled tree shapes compatible with a labeled binary perfect phylogeny. Each row of Fig. 8C corresponds to one of the unranked labeled tree shapes compatible with the labeled perfect phylogeny.

#### Definition 17

(*Unranked labeled tree shape* $T^{X}_{n}$ *compatible with a labeled perfect phylogeny* $\pi \in \Pi ^{L}_{n}$). An unranked labeled tree shape with *n* leaves, $T^{X}_{n}$, is compatible with a perfect phylogeny $\pi \in \Pi ^{L}_n$ if the unranked unlabeled tree shape $T_{n}$ obtained by removing the labels from ${T}^{X}_{n}$ is compatible with $\pi$ and the one-to-one correspondence between the *k* leaves of $\pi$ and the *k* disjoint subtrees of $T^{X}_{n}$ correspond to the same partition of the individual sequences.

#### Proposition 18

For $n_1, n_2 \ge 1$ and $\pi _{1}\in \Pi _{n_{1}}^L, \pi _{2}\in \Pi _{n_{2}}^L$, the number of unranked labeled tree shapes compatible with a labeled binary perfect phylogeny $\pi =(\pi _{1},\pi _{2})$ is

20 $$\begin{aligned} \big |{\mathcal {G}}^{X}_{c}(\pi )\big |= \big |{\mathcal {G}}^{X}_{c}(\pi _{1})\big | \, \big |{\mathcal {G}}^{X}_{c}(\pi _{2})\big |. \end{aligned}$$

#### Proof

We divide $\pi$ at the root into two subtrees, one with $n_1$ leaves and perfect phylogeny $\pi _1$, and the other with $n_2$ leaves and perfect phylogeny $\pi _2$. The subtrees must partition the sampled sequences in the same way as $\pi$. The number of such trees is the simply product of the numbers of unranked labeled trees for the two subtrees. As in Proposition 15, perfect phylogenies $\pi _{1}$ and $\pi _{2}$ are not identical because they correspond to different sets of sequences; with the ranking dropped, unlike in Proposition 15, we need not consider the number of ways of interleaving the internal nodes of the two subtrees. $\square$

For some of the perfect phylogenies of 10 taxa (with an arbitrary labeling), counts for the number of unranked labeled tree shapes appear in Table 1. The number of unranked labeled tree shapes far exceeds the number of unranked unlabeled tree shapes, and it generally exceeds the number of ranked unlabeled tree shapes.

For the example ((4,2),6), the number of unranked labeled tree shapes compatible with this (arbitrarily labeled) perfect phylogeny is $|{\mathcal {G}}_c^X((4,2))| \, |{\mathcal {G}}_c^X((6))| = |{\mathcal {G}}_c^X((4))| \,|{\mathcal {G}}_c^X((2))| \,|{\mathcal {G}}_c^X((6))| = X_4 X_2 X_6 = 15 \times 1 \times 945 =14{,}175$.

For binary labeled perfect phylogenies $B_x(n)=(x,n-x)$, the one compatible with the largest number of unranked labeled tree shapes follows the result of Proposition 16. Let $b_x''(n)$ denote the number of unranked labeled tree shapes compatible with $B_x(n)$.

#### Proposition 19

Fix $n \ge 2$. Among the values $b_1''(n), b_2''(n), \ldots , b_{\lfloor \frac{n}{2} \rfloor }''(n)$, the largest is $b_1''(n)$, and the smallest is $b_{\lfloor \frac{n}{2} \rfloor }''(n)$.

#### Proof

Applying Proposition 18, we have $b_x''(n)= X_x \, X_{n-x}$ for $1 \le x \le \lfloor \frac{n}{2} \rfloor$. Simplifying with Eq. 2, we obtain

$$\begin{aligned} b_x''(n) = \frac{(n-2)!}{2^{n-2}} \frac{{2x-2 \atopwithdelims ()x-1}{2n-2x-2 \atopwithdelims ()n-x-1}}{{n-2 \atopwithdelims ()x-1}}. \end{aligned}$$

Then $b_{x+1}''(n)/b_{x}''(n) = \frac{2x-1}{2n-2x-3} \le 1$ for $1 \le x \le \frac{n-1}{2}$, with equality requiring $x=\frac{n-1}{2}$, so that $b_{x}''(n)$ monotonically decreases from $x=1$ to $x=\lfloor \frac{n}{2} \rfloor$. $\square$

In Table 1, we observe that as in Proposition 19, $b_1''(10)=2{,}027{,}025$ exceeds $b_2''(10)$, $b_3''(10)$, $b_4''(10)$, and $b_5''(10)$, among which $b_5''(10)=11{,}025$ is the smallest.

## Enumeration for multifurcating perfect phylogenies

Recall that perfect phylogenies need not be strictly binary, and that nodes can have more than two descendants. To complete the description of the numbers of trees of various types that are compatible with a perfect phylogeny, we must consider multifurcating perfect phylogenies. We proceed by reducing the multifurcating case to the binary case that has already been solved.

**Fig. 9:**
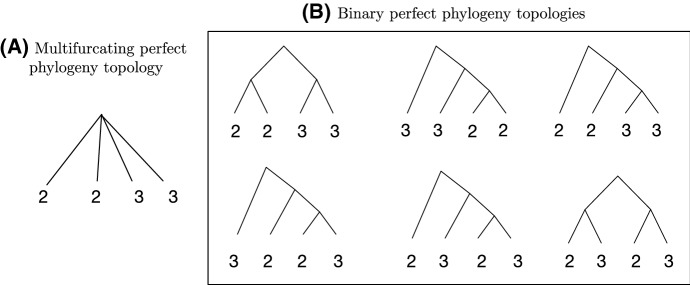
Example of all possible binary perfect phylogeny topologies for a given multifurcating perfect phylogeny topology. The binary perfect phylogenies are obtained from a multifurcating perfect phylogeny by resolving multifurcating nodes into sequences of bifurcations

We now consider a **multifurcating perfect phylogeny** that consists of a single internal node subtending *k* leaves with labels $n_{1},n_{2},\ldots ,n_{k}$. An example is depicted in Fig. 9. Because multiple leaves can each correspond to groups with the same number of taxa, so that the same numerical label can be assigned to many of those leaves, it is convenient to denote the vector of unique labels by ${\mathbf {a}}=(a_{1},a_{2},\ldots ,a_{s})$ and the corresponding vector of their multiplicities by ${\mathbf {m}}=(m_{1},m_{2},\ldots ,m_{s})$, where $m_{j}$ denotes the number of leaves with label $a_{j}$, $1 \le j \le s \le k$. In the example of Fig. 9, ${\mathbf {a}}=(2,3)$ and ${\mathbf {m}}=(2,2)$, as two leaves $(m_1=2)$ have label 2 $(a_1=2)$ and two leaves $(m_2=3)$ have label 3 $(a_2=3)$.

We extend the notion of the binary perfect phylogeny poset to the multifurcating case. We define $\pi \le \sigma$ for two multifurcating perfect phylogenies if $\sigma$ can be obtained by sequentially collapsing pairs of pendant edges of $\pi$. Given two multifurcating perfect phylogenies $\pi _{1}$ and $\pi _{2}$, their meet $\pi _{1} \wedge \pi _{2}$ is the largest multifurcating perfect phylogeny that refines both $\pi _{1}$ and $\pi _{2}$. For example, the meet between $\pi _{1}=(1,2,3,(2,2))$ and $\pi _{2}=(1,2,2,(2,3))$ is given by:

$$\begin{aligned} (1,2,3,(2,2)) \wedge (1,2,2,(2,3))&= (1,(2,2),(2,3)). \end{aligned}$$

Similarly, their join is the smallest multifurcating perfect phylogeny $\pi _{1} \vee \pi _{2}$ for which both $\pi _{1}$ and $\pi _{2}$ are refinements:

$$\begin{aligned} (1,2,3,(2,2)) \vee (1,2,2,(2,3))&= (1,2,2,2,3). \end{aligned}$$

The lattice structure enables us to count the number of ranked unlabeled tree shapes compatible with a multifurcating perfect phylogeny $\pi =(n_{1},n_{2},\ldots ,n_{k})$. We use a recursive inclusion-exclusion principle with label vector ${\mathbf {a}}$ and multiplicities ${\mathbf {m}}$. The key idea is to decompose the computation into a sum over all possible binary perfect phylogenies, applying Propositions 10 and 11 to each binary perfect phylogeny. To recursively generate all possible binary perfect phylogenies from $\pi$, we define the operator ${\mathcal {B}}_{i,j}(\pi )$ that collapses two leaves with labels $a_{i}$ and $a_{j}$ in $\pi$. For example ${\mathcal {B}}_{2,3}(2,2,3,4)=((2,3),2,4)$. If $\sum ^{s}_{i=1} m_{i}>2$, then

21 $$\begin{aligned} |{\mathcal {G}}_{c}(\pi )|&=\underbrace{\sum _{i=1}^s|{\mathcal {G}}_{c}({\mathcal {B}}_{i,i}(\pi ))|\,1_{m_{i}>1}}_{\small {\begin{array}{c} \text {collapsing two pendant edges}\\ \text {with the same leaf values} \end{array}}}+\underbrace{\sum _{i=1}^{s-1} \sum _{j=i+1}^s|{\mathcal {G}}_{c}({\mathcal {B}}_{i,j}(\pi ))\,|1_{m_{i}>0} \, 1_{m_{j}>0}}_{\small {\begin{array}{c} \text {collapsing two pendant edges}\\ \text {with different leaf values} \end{array}}} \nonumber \\&\quad - \underbrace{\sum _{i=1}^{s-1} \sum _{j=i+1}^s|{\mathcal {G}}_{c}({\mathcal {B}}_{i,i}(\pi ) \wedge {\mathcal {B}}_{j,j}(\pi ))|\,1_{m_{i}>1}\,1_{m_{j}>1}}_{\small {\begin{array}{c} \text {collapsing all pairs containing two distinct pairs of pendant edges,}\\ \text {each pair with the same leaf values} \end{array}}} \nonumber \\&\quad - \underbrace{\sum _{i=1}^{s-1} \sum _{j=i+1}^s \sum _ {\begin{array}{c} {k=1}\\ {k \ne i, k \ne j} \end{array}} ^s |{\mathcal {G}}_{c}({\mathcal {B}}_{i,j}(\pi ) \wedge {\mathcal {B}}_{k,k}(\pi )) | \, 1_{m_{i}>0} \, 1_{m_{j}>0} \, 1_{m_{k}>1}}_{\small {\begin{array}{c} \text {collapsing a pair of edges with different leaf values}\\ \text {and collapsing a pair of edges with the same leaf values} \end{array}}} \nonumber \\&\quad - \underbrace{\sum _{i=1}^{s-1} \sum _{j=i+1}^s \sum _ {\begin{array}{c} {k=1}\\ {k \ne i, k \ne j} \end{array}} ^{s-1} \sum _ {\begin{array}{c} {\ell =k+1 }\\ {\ell \ne i, \ell \ne j} \end{array}}^s |{\mathcal {G}}_{c}({\mathcal {B}}_{i,j}(\pi ) \wedge {\mathcal {B}}_{k,\ell }(\pi ))|\, 1_{m_{i}>0}\, 1_{m_{j}>0}\, 1_{m_{k}>0}\, 1_{m_{\ell }>0}}_{\small {\begin{array}{c} \text {collapsing two different pairs of pendant edges,}\\ \text {each pair with different leaf values} \end{array}}}. \end{aligned}$$

To interpret Eq. 21 as an inclusion-exclusion formula, notice that the first two sums that are added on the right-hand side of Eq. 21 correspond to enumerations of single events (so that the sum is analogous to a union $\cup A_{i}$), and the following three sums that are subtracted correspond to intersections of pairs of these events (analogous to intersections $A_{i} \cap A_{j}$).

Equation 21 provides a recursive approach for counting the number of ranked unlabeled tree shapes compatible with a multifurcating perfect phylogeny by expressing the calculation in terms of binary perfect phylogenies. The recursive application of the equation proceeds until all terms reach $\sum ^{s}_{i=1}m_{i}=2$, when the binary perfect phylogenies are reached.

### Example 20

The number of ranked unlabeled tree shapes compatible with $\pi =(2,2,3,3)$ is:

$$\begin{aligned} |{\mathcal {G}}^{T}_{c}(2,2,3,3)|&=|{\mathcal {G}}^{T}_{c}((2,2),3,3)| +|{\mathcal {G}}^{T}_{c}(2,2,(3,3))| \\&\quad \quad +|{\mathcal {G}}^{T}_{c}((2,3),2,3)| -|{\mathcal {G}}^{T}_{c}((2,2),(3,3))|\\&\quad = \big [ |{\mathcal {G}}^{T}_{c}((2,2),(3,3))| +|{\mathcal {G}}^{T}_{c}(((2,2),3),3)| \big ]\\&\quad \quad + \big [ |{\mathcal {G}}^{T}_{c}((2,2),(3,3))| +|{\mathcal {G}}^{T}_{c}(((3,3),2),2)| \big ]\\&\quad \quad + \big [ |{\mathcal {G}}^{T}_{c}(((2,3),2),3)| +|{\mathcal {G}}^{T}_{c}(((2,3),3),2)| \\&\quad \quad + |{\mathcal {G}}^{T}_{c}((2,3),(2,3))| \big ] -|{\mathcal {G}}^{T}_{c}((2,2),(3,3))| \\&\quad =|{\mathcal {G}}^{T}_{c}((2,2),(3,3))| +|{\mathcal {G}}^{T}_{c}(((2,2),3),3)| \\&\quad \quad +|{\mathcal {G}}^{T}_{c}(((3,3),2),2)| +|{\mathcal {G}}^{T}_{c}(((2,3),2),3)| \\&\quad \quad +|{\mathcal {G}}^{T}_{c}(((2,3),3),2)| + |{\mathcal {G}}^{T}_{c}((2,3),(2,3))| \\&\quad =168+280+144+420+360+315=1687. \end{aligned}$$

In obtaining this sum, in intermediate steps, we use the fact that the values of ${\mathcal {G}}_{c}^T$ for (2), (3), (2,2), (3,3), (2,3), ((2,2),3), ((3,3),2), ((2,3),2)), and (2,3),3) are 1, 1, 1, 3, 3, 10, 18, 15, and 45, respectively.

For counting the number of unranked unlabeled tree shapes compatible with $\pi =(n_{1},n_{2},\ldots ,n_{k})$, we simply replace ${\mathcal {G}}^{T}_{c}$ with ${\mathcal {G}}_{c}$ in Eq. 21. We use Propositions 7 and 8 in place of Propositions 10 and 11.

### Example 21

The number of unranked unlabeled tree shapes compatible with $\pi =(2,2,3,3)$ is:

$$\begin{aligned} |{\mathcal {G}}_{c}(2,2,3,3)|&= |{\mathcal {G}}_{c}((2,2),(3,3))|+|{\mathcal {G}}_{c}(((2,2),3),3)|+|{\mathcal {G}}_{c}(((3,3),2),2)|\\&\quad +|{\mathcal {G}}_{c}(((2,3),2),3)|+|{\mathcal {G}}_{c}(((2,3),3),2)|+ |{\mathcal {G}}_{c}((2,3),(2,3))| \\&=1+1+1+1+1+1=6. \end{aligned}$$

This example is quite straightforward; the values of ${\mathcal {G}}_{c}$ for the perfect phylogenies that appear in intermediate steps—(2), (3), (2,2), (3,3), (2,3), ((2,2),3), ((3,3),2), ((2,3),2)), and ((2,3),3)—all equal 1.

To count the number of ranked labeled tree shapes compatible with a labeled multifurcating perfect phylogeny $\pi =(n_{1},n_{2},\ldots ,n_{k})$, we assume that although any leaf in the perfect phylogeny can have multiplicity larger than one, each leaf is uniquely defined by its associated taxa, all of which are all assumed to have different labels. Therefore, we take ${\mathbf {a}}=(n_{1},n_{2},\ldots ,n_{k})$ and ${\mathbf {m}}=(1,1,\ldots ,1)$. Equation 21 reduces to

22 $$\begin{aligned} |{\mathcal {G}}^{L}_{c}(\pi )|&=\underbrace{\sum _{i=1}^{s-1} \sum _{j=i+1}^s|{\mathcal {G}}^{L}_{c}({\mathcal {B}}_{i,j}(\pi ))|\, 1_{m_{i}>0}\, 1_{m_{j}>0}}_{\small {\begin{array}{c} \text {collapsing two pendant edges} \end{array}}} \nonumber \\&\quad - \underbrace{\sum _{i=1}^{s-1} \sum _{j=i+1}^s \sum _ {\begin{array}{c} {k=1}\\ {k \ne i, k \ne j} \end{array}} ^{s-1} \sum _ {\begin{array}{c} {\ell =k+1}\\ {\ell \ne i, \ell \ne j} \end{array}} ^s|{\mathcal {G}}^{L}_{c}({\mathcal {B}}_{i,j}(\pi ) \wedge {\mathcal {B}}_{k,\ell }(\pi ))|\, 1_{m_{i}>0}\, 1_{m_{j}>0}\, 1_{m_{k}>0}\, 1_{m_{\ell }>0}}_{\small {\begin{array}{c} \text {collapsing two pairs of pendant edges} \end{array}}}. \end{aligned}$$

The enumeration makes use of Proposition 15.

### Example 22

Consider a labeled multifurcating perfect phylogeny that groups 2, 2, 3, and 3 taxa at the root. We assume that ${\mathbf {a}}=(a_{1},a_{2},a_{3},a_{4})=(2,2,3,3)$. Applying the recursion formula in Eq. 22, we get

$$\begin{aligned}&|{\mathcal {G}}^{L}_{c}(a_{1},a_{2},a_{3},a_{4})|\\&= |{\mathcal {G}}^{L}_{c}((a_{1},a_{2}),a_{3},a_{4})|+|{\mathcal {G}}^{L}_{c}((a_{1},a_{3}),a_{2},a_{4})|+|{\mathcal {G}}^{L}_{c}((a_{1},a_{4}),a_{2},a_{3})|\\&\quad + |{\mathcal {G}}^{L}_{c}((a_{2},a_{3}),a_{1},a_{4})|+|{\mathcal {G}}^{L}_{c}((a_{2},a_{4}),a_{1},a_{3})|+|{\mathcal {G}}^{L}_{c}((a_{3},a_{4}),a_{1},a_{2})|\\&\quad - |{\mathcal {G}}^{L}_{c}((a_{1},a_{2}),(a_{3},a_{4}))|-|{\mathcal {G}}^{L}_{c}((a_{1},a_{3}),(a_{2},a_{4}))|-|{\mathcal {G}}^{L}_{c}((a_{1},a_{4}),(a_{2},a_{3}))|\\&=|{\mathcal {G}}^{L}_{c}((2,2),3,3)|+4|{\mathcal {G}}^{L}_{c}((2,3),2,3)|+|{\mathcal {G}}^{L}_{c}((3,3),2,2)|\\&\quad -|{\mathcal {G}}^{L}_{c}((2,2),(3,3))|-2|{\mathcal {G}}^{L}_{c}((2,3),(2,3))|. \end{aligned}$$

Now, because

$$\begin{aligned} |{\mathcal {G}}^{L}_{c}(a_{1},a_{2},a_{3})|&= |{\mathcal {G}}^{L}_{c}((a_{1},a_{2}),a_{3})|+|{\mathcal {G}}^{L}_{c}((a_{1},a_{3}),a_{2})|+|{\mathcal {G}}^{L}_{c}((a_{2},a_{3}),a_{1})|, \end{aligned}$$

we have

$$\begin{aligned} |{\mathcal {G}}^{L}_{c}((2,2),3,3)|&= 2|{\mathcal {G}}^{L}_{c}(((2,2),3),3)|+|{\mathcal {G}}^{L}_{c}((2,2),(3,3))| \\ |{\mathcal {G}}^{L}_{c}((2,3),2,3)|&= |{\mathcal {G}}^{L}_{c}(((2,3),2),3)|+|{\mathcal {G}}^{L}_{c}(((2,3),3),2)|+|{\mathcal {G}}^{L}_{c}((2,3),(2,3))| \\ |{\mathcal {G}}^{L}_{c}((3,3),2,2)|&= 2|{\mathcal {G}}^{L}_{c}(((3,3),2),2)|+|{\mathcal {G}}^{L}_{c}((2,2),(3,3))|. \end{aligned}$$

Summing all terms, we get

$$\begin{aligned}&|{\mathcal {G}}^{L}_{c}(a_{1},a_{2},a_{3},a_{4})| \\ {}&=2|{\mathcal {G}}^{L}_{c}(((2,2),3),3)|+2|{\mathcal {G}}^{L}_{c}(((3,3),2),2)|+|{\mathcal {G}}^{L}_{c}((2,2),(3,3))|\\&\quad +4|{\mathcal {G}}^{L}_{c}(((2,3),2),3)|+4|{\mathcal {G}}^{L}_{c}(((2,3),3),2)|+2|{\mathcal {G}}^{L}_{c}((2,3),(2,3))|\\&=2\times 5040 + 2 \times 2592 + 6048 + 4 \times 3780 + 4 \times 3240 + 2 \times 5670= 60{,}732. \end{aligned}$$

In obtaining this sum, we use the fact that the values of ${\mathcal {G}}_{c}^L$ for (2), (3), (2,2), (3,3), (2,3), ((2,2),3), ((3,3),2), ((2,3),2)), and ((2,3),3), and are 1, 3, 2, 54, 9, 60, 324, 45, and 405, respectively.

The number of unranked labeled tree shapes compatible with $\pi =(n_{1},n_{2},\ldots ,n_{k})$ is obtained by replacing ${\mathcal {G}}^{L}_{c}$ with ${\mathcal {G}}^{X}_{c}$ in Eq. 22. We use Proposition 18 in place of Proposition 15.

### Example 23

The number of unranked labeled tree shapes compatible with a labeled multifurcating perfect phylogeny that groups 2, 2, 3, and 3 taxa at the root, with ${\mathbf {a}}=(a_{1},a_{2},a_{3},a_{4})=(2,2,3,3)$ is:

$$\begin{aligned}&|{\mathcal {G}}^{X}_{c}(a_{1},a_{2},a_{3},a_{4})|\\&=2|{\mathcal {G}}^{X}_{c}(((2,2),3),3)|+2|{\mathcal {G}}^{X}_{c}(((3,3),2),2)|+|{\mathcal {G}}^{X}_{c}((2,2),(3,3))|\\&\quad +4|{\mathcal {G}}^{X}_{c}(((2,3),2),3)|+4|{\mathcal {G}}^{X}_{c}(((2,3),3),2)|+2|{\mathcal {G}}^{X}_{c}((2,3),(2,3))|\\&=2\times 9 + 2 \times 9 + 9 + 4 \times 9 + 4 \times 9 + 2 \times 9= 135. \end{aligned}$$

The sum uses values of ${\mathcal {G}}_{c}^{X}$ for (2), (3), (2,2), (3,3), (2,3), ((2,2),3), ((3,3),2), ((2,3),2)), and ((2,3),3), equal to 1, 3, 1, 9, 3, 3, 9, 3, and 9, respectively.

**Fig. 10:**
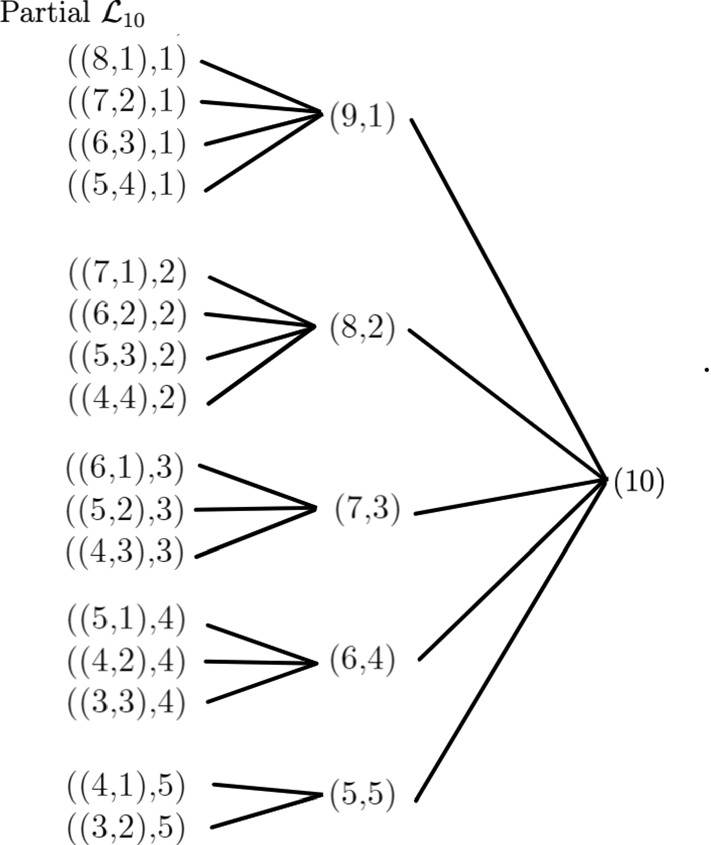
Partial Hasse diagram of the lattice of binary perfect phylogenies with $n=10$ taxa. Only those perfect phylogenies appearing in Table 1 are shown

The entries in the table are obtained by repeated use of Propositions 7 and 8 for unranked unlabeled tree shapes, Propositions 10 and 11 for ranked unlabeled tree shapes, Proposition 15 for ranked labeled tree shapes, and Proposition 18 for unranked labeled tree shapes. An arbitrary labeling of the perfect phylogeny is assumed for counting the associated ranked and unranked labeled tree shapes. Figure 10 shows the corresponding partial Hasse diagram of the lattice of binary perfect phylogenies with 10 taxa.

**Table 1:** Number of trees compatible with example perfect phylogenies of 10 taxa

Perfect phylogeny	Unranked unlabeled tree shapes	Ranked unlabeled tree shapes	Ranked labeled tree shapes	Unranked labeled tree shapes
(9,1)	46	1385	57,153,600	2,027,025
(8,2)	23	2176	12,700,800	135,135
(7,3)	11	1708	4,762,800	31,185
(6,4)	12	1792	2,721,600	14,175
(5,5)	6	875	2,268,000	11,025
((8,1),1)	23	272	1,587,600	135,135
((7,2),1)	11	427	396,900	10,395
((6,3),1)	6	336	170,100	2835
((5,4),1)	6	350	113,400	1575
((7,1),2)	11	488	453,600	10,395
((6,2),2)	6	768	129,600	945
((5,3),2)	3	600	64,800	315
((4,4),2)	3	320	51,840	225
((6,1),3)	6	448	226,800	2835
((5,2),3)	3	700	75,600	315
((4,3),3)	2	560	45,360	135
((5,1),4)	6	560	181,440	1575
((4,2),4)	4	896	72,576	225
((3,3),4)	2	336	54,432	135
((4,1),5)	5	560	226,800	1575
((3,2),5)	3	735	113,400	315

## Conclusion

The infinitely-many-sites mutations model is a popular model of molecular variation for problems of population genetics (Wakeley 2008) and related areas (Jones et al. 2020), in which constraints are imposed on the space of trees that can explain the observed patterns of molecular variation. A realization of the coalescent model on a genealogy and a superimposed infinitely-many-sites mutation model can be summarized as a perfect phylogeny. Here, we have examined combinatorial properties of the genealogical tree structures that are compatible with a perfect phylogeny, demonstrating that the binary perfect phylogenies possess a lattice structure (Theorem 5). We have used this lattice structure to provide recursive enumerative results counting the trees—unranked unlabeled trees, ranked unlabeled trees, ranked labeled trees, and unranked labeled trees—compatible with binary perfect phylogenies. Further, for multifurcating perfect phylogenies, we have exploited a recursive inclusion-exclusion principle to decompose a multifurcating perfect phylogeny into all possible binary perfect phylogenies, extending the utility of our lattice approach from bifurcating structures to more general structures.

In our enumerative results, the count of the number of trees of a specified type that are compatible with a perfect phylogeny is obtained by a decomposition of the perfect phylogeny at its root. The number of associated trees is obtained by counting trees for each subtree immediately descended from the root of the perfect phylogeny—and where appropriate, counting interleavings of nodes within those trees, taking care to consider cases that avoid double-counting, or both. This same technique was applicable for each of the types of trees we considered, appearing in Sects. 3.2, 3.3, 3.4, 3.5, and 4. We have provided examples for relatively small cases with $n=10$ taxa (Table 1, Fig. 10). Owing to the recursive structure of the computation, the decomposition itself proceeds rapidly from the root through the internal nodes, so that a count can be quickly obtained even if the number itself is large. Our algorithmic implementation in python does have a computational precision limitation, but it accommodates numbers up to the order of $10^{290}$.

We obtained results concerning the cherry perfect phylogenies with the largest numbers of ranked unlabeled, unranked labeled, and ranked labeled tree shapes (Propositions 12, 16, and 19), and it will be informative to seek a similar result for the unranked unlabeled case. The result in Proposition 12 on asymptotic growth of the number of ranked unlabeled tree shapes compatible with a binary perfect phylogeny is reminiscent of a result concerning “lodgepole” trees. A number of studies have examined another combinatorial structure for evolutionary trees, the number of “coalescent histories” associated with a labeled species tree and its matching labeled gene tree. These coalescent histories encode different evolutionary scenarios possible for the coalescence of gene lineages on a species tree. Disanto and Rosenberg (2015) found that the lodgepole trees, a class of trees in which cherry nodes with 2 descendants successively branch from a single species tree edge, possesses a particularly large number of coalescent histories. Similarly, in Proposition 12, as *n* increases, the number of ranked unlabeled tree shapes compatible with a cherry perfect phylogeny is largest when the perfect phylogeny has one subtree with sample size 2.

Perfect phylogenies have been widely studied in varied estimation problems, for the “perfect phylogeny problem” asking whether a perfect phylogeny can be constructed from data given on a set of characters (Agarwala and Fernández-Baca 1993; Kannan and Warnow 1997; Felsenstein 2004; Gusfield 2014; Steel 2016), statistical inference of evolutionary parameters under the coalescent (Griffiths and Tavaré 1994; Stephens and Donnelly 2000; Tavaré 2004; Palacios et al. 2019; Cappello et al. 2020b), and algorithmic estimation of haplotype phase from diploid data (Gusfield 2002; Bafna et al. 2004; Gusfield 2014). However, the literature on perfect phylogenies has largely focused on such applications and on algorithmic problems of obtaining perfect phylogenies from data under various constraints, with little emphasis on the enumerative combinatorics of the perfect phylogenies themselves, and of their associated refinements. In describing a lattice for the binary perfect phylogenies with sample size *n*, this study suggests that the mathematical properties of sets of perfect phylogenies as combinatorial structures *per se* can be informative. The link to coalescent histories suggests possible connections to related concepts such as “ancestral configurations” (Wu 2012; Disanto and Rosenberg 2017), which also can be described in terms of lattices (Alimpiev and Rosenberg 2022); it will be useful to consider perfect phylogenies alongside such structures arising in the combinatorics of evolutionary trees.

**Table 2:** Ratio of the number of unranked labeled and unranked unlabeled tree shapes and ratio of the number of ranked labeled and ranked unlabeled tree shapes compatible with three perfect phylogenies of 10, 20 and 50 taxa

Perfect phylogeny	Ratio for unranked tree shapes	Ratio for ranked tree shapes
(5,5)	1837.5	2592
(15,5)	$\sim 1.54\times 10^{12}$	$\sim 1.26\times 10^{12}$
(45,5)	$\sim 2.17\times 10^{53}$	$\sim 8.18\times 10^{52}$

Finally, returning to considerations of coalescent-based inference from sequences, recall that inference of evolutionary parameters from a given perfect phylogeny is performed by integrating over the space of genealogies. A standard approach to inference integrates over the space of ranked labeled tree shapes generated by the Kingman coalescent (Drummond et al. 2012). However, this inference is computationally intractable for large sample sizes. We have observed a striking reduction in the cardinality of the set of ranked (and unranked) unlabeled tree shapes compatible with an observed perfect phylogeny, relative to the number of ranked (and unranked) labeled tree shapes compatible with an observed perfect phylogeny (Tables 1 and 2). This observation contributes to a growing branch of the area of coalescent-based inference (Sainudiin et al. 2015; Palacios et al. 2015, 2019; Cappello et al. 2020a) that can make use of ranked unlabeled trees to estimate the evolutionary parameters.

## Appendix: Proof of Theorem 5

To prove Theorem 5, we must verify four pairs of conditions concerning perfect phylogenies $\pi \in \Pi _{n} \cup \{\emptyset \}$. Note that any binary perfect phylogeny $\pi \in \Pi _{n} \cup \{\emptyset \}$ is equal to $\emptyset$, (*n*), or $(\pi _1,\pi _2)$ for two non-empty binary perfect phylogenies $\pi _1 \in \Pi _{n_1}$ and $\pi _2 \in \Pi _{n_2}$, where $1 \le n_1,n_2 < n$ and $n_1+n_2=n$. Hence, we must demonstrate the four pairs of conditions for perfect phylogeny pairs that include $\emptyset$, (*n*), or both, and for perfect phylogeny pairs that include neither $\emptyset$ nor (*n*).

Because perfect phylogenies can be decomposed into smaller perfect phylogenies, we proceed by induction on *n*, with a base case of $n=1$. In the inductive step we assume that $(\Pi _{k} \cup \{\emptyset \}, \wedge , \vee )$ is a lattice for all *k*, $1 \le k < n$. We then verify that it follows that $(\Pi _{n} \cup \{\emptyset \}, \wedge , \vee )$ is a lattice. We start with Condition 2, which is trivial.

### Condition 2: $\pi \wedge \sigma = \sigma \wedge \pi$ and $\pi \vee \sigma = \sigma \vee \pi$

For all *n*, condition 2 of the definition of a lattice is trivially satisfied, as the operations $\wedge$ and $\vee$ are symmetric by definition. In subsequent derivations, we frequently apply Condition 2 without always noting its application.

### The $n=1$ case for conditions 1, 3, and 4

Consider $n=1$, for which $\Pi _1$ contains only the perfect phylogeny (1), and $\Pi _1 \cup \{\emptyset \}$ contains only (1) and $\emptyset$. For $\Pi _1 \cup \{\emptyset \}$, demonstrating Condition 1 of the requirements for a lattice requires that we show $(1) \wedge (1) = (1)$, $\emptyset \wedge \emptyset = \emptyset$, $(1) \vee (1) = (1)$, and $\emptyset \vee \emptyset = \emptyset$. These four relations are true by parts (3), (1), (4), and (2) of Definition 1, respectively.

Demonstrating Condition 3 requires that we verify a pair of conditions for each of the eight choices of (*x*, *y*, *z*) for $x,y,z \in \Pi _1 \cup \{\emptyset \}$. Demonstrating Condition 4 requires that we verify a pair of conditions for each of the four choices of (*x*, *y*). The 16 verifications for Condition 3 and eight verifications for Condition 4 all quickly follow by Definition 1 (1–4). Hence, $(\Pi _{1} \cup \{\emptyset \}, \wedge , \vee )$ is a lattice.

### Condition 1: $\pi \wedge \pi = \pi$ and $\pi \vee \pi = \pi$

First, we demonstrate the first part of the condition. We see $\emptyset \wedge \emptyset = \emptyset$ by Definition 1 (1) and $(n) \wedge (n) = (n)$ by Definition 1 (3).

Consider $\pi = (\pi _1,\pi _2)$ for $\pi _1 \in \Pi _{n_1}$ and $\pi _2 \in \Pi _{n_2}$, where $1 \le n_1,n_2 < n$ and $n_1 + n_2 = n$.

$$\begin{aligned} \pi \wedge \pi&= (\pi _1,\pi _2) \wedge (\pi _1,\pi _2) \\&= (\pi _1 \wedge \pi _1, \pi _2 \wedge \pi _2) \vee (\pi _1 \wedge \pi _2, \pi _2 \wedge \pi _1) \text { by Prop.~2 (2)}\\&= (\pi _1, \pi _2) \vee (\pi _1 \wedge \pi _2, \pi _1 \wedge \pi _2) \text { by the inductive hypothesis}. \end{aligned}$$

If $n_1 \ne n_2$, then we apply Definition 1 (5), the convention $(\pi , \emptyset ) = \emptyset$, and Definition 1 (2), and we obtain $\pi \wedge \pi = (\pi _1,\pi _2) \vee (\emptyset , \emptyset ) = (\pi _1, \pi _2) \vee \emptyset = (\pi _1, \pi _2) = \pi$. If $n_1 = n_2$, then we have two cases: $\pi _1 \le \pi _2$ (without loss of generality), and $\pi _1,\pi _2$ are not comparable.

If $\pi _1 \le \pi _2$, then $\pi _1 \wedge \pi _2 = \pi _1$ and $\pi _1 \vee \pi _2 = \pi _2$. By Proposition 2 (3), $(\pi _1,\pi _2) \vee (\pi _1,\pi _1) = (\pi _1, \pi _2 \vee \pi _1) = (\pi _1,\pi _2) = \pi$, so that $\pi \wedge \pi = \pi$.

If $\pi _1$ and $\pi _2$ are not comparable, then by Definition 1 (11), $\pi _{1}\wedge \pi _{2}=\delta$ for some $\delta \in (\Pi _{n1} \cup \{\emptyset \}) {\setminus } \{\pi _{1},\pi _{2}\}$, with $\delta \vee \pi _{1}=\pi _{1}$ and $\delta \vee \pi _{2}=\pi _{2}$. We then have by Proposition 2 (3),

$$\begin{aligned} (\pi _1, \pi _2) \vee (\pi _1 \wedge \pi _2, \pi _1 \wedge \pi _2)&= (\pi _1, \pi _2) \vee (\delta ,\delta ). \end{aligned}$$

But $(\delta ,\delta )$ refines $(\pi _{1},\pi _{2})$, as $\delta$ refines $\pi _{1}$ and $\delta$ refines $\pi _{2}$, so that $(\pi _1, \pi _2)$ can be obtained by collapsing cherries separately in the two subtrees of $(\delta , \delta )$. Hence, $\pi \wedge \pi = (\pi _1, \pi _2) \vee (\delta , \delta ) = (\pi _1, \pi _2) = \pi$.

For the second part of the condition, we have $\emptyset \vee \emptyset = \emptyset$ by Definition 1 (2) and $(n) \vee (n) = (n)$ by Definition 1 (4). Consider $\pi = (\pi _1,\pi _2)$ for $\pi _1 \in \Pi _{n_1}$ and $\pi _2 \in \Pi _{n_2}$, where $1 \le n_1,n_2 < n$ and $n_1 + n_2 = n$.

$$\begin{aligned} \pi \vee \pi&= (\pi _1,\pi _2) \vee (\pi _1,\pi _2) \\&= (\pi _1, \pi _2 \vee \pi _2) \text { by Prop.~2 (3)}\\&= (\pi _1, \pi _2) \text { by the inductive hypothesis} \\&= \pi . \end{aligned}$$

### Condition 4: $\pi \wedge (\pi \vee \sigma )=\pi$ and $\pi \vee (\pi \wedge \sigma )=\pi$

First, we see that both parts of the condition hold if at least one of $\pi , \sigma$ is in $\{ \emptyset ,(n)\}$, by Definition 1 (1–4). Next, we have the following 3 cases:

i. If $\pi \le \sigma$, then $\pi \wedge \sigma = \pi$ and $\pi \vee \sigma = \sigma$. Hence, $\pi \wedge (\pi \vee \sigma )=\pi \wedge \sigma = \pi$. By Condition 1, $\pi \vee (\pi \wedge \sigma )=\pi \vee \pi =\pi$.
ii. If $\sigma \le \pi$, then $\pi \wedge \sigma =\sigma$ and $\pi \vee \sigma =\pi$. Hence, by Condition 1, $\pi \wedge (\pi \vee \sigma )=\pi \wedge \pi = \pi$. We also have $\pi \vee (\pi \wedge \sigma )=\pi \vee \sigma =\pi$.
iii. If $\pi$ and $\sigma$ are not comparable, then by Proposition 2 (5), there exists a perfect phylogeny $\gamma$ such that $\pi \vee \sigma = \gamma$, $\pi \wedge \gamma =\pi$, and $\sigma \wedge \gamma =\sigma$. Hence $\pi \wedge (\pi \vee \sigma )=\pi \wedge \gamma = \pi$. By Proposition 2 (5), there exists a perfect phylogeny $\rho$ such that $\pi \wedge \sigma =\rho$, $\pi \vee \rho =\pi$, and $\sigma \vee \rho = \sigma$. We have $\pi \vee (\pi \wedge \sigma )=\pi \vee \rho =\pi$.

### Condition 3: $\pi \wedge (\sigma \wedge \rho )=(\pi \wedge \sigma ) \wedge \rho$ and $\pi \vee (\sigma \vee \rho )=(\pi \vee \sigma ) \vee \rho$

First, we see that both parts of the condition hold if at least one of $\pi , \sigma , \rho$ is in $\{\emptyset ,(n)\}$, by Definition 1 (1–4). Assume now that $\pi =(\pi _{1},\pi _{2})$, $\sigma =(\sigma _{1},\sigma _{2})$, and $\rho =(\rho _{1},\rho _{2})$. Then

$$\begin{aligned}&\pi \wedge (\sigma \wedge \rho )\\&=(\pi _{1},\pi _{2}) \wedge \left( (\sigma _{1},\sigma _{2}) \wedge (\rho _{1},\rho _{2}) \right) \\&=(\pi _{1},\pi _{2}) \wedge [ (\sigma _{1} \wedge \rho _{1}, \sigma _{2}\wedge \rho _{2}) \vee (\sigma _{1} \wedge \rho _{2}, \sigma _{2}\wedge \rho _{1})] \text { by Prop.~2 (2)}\\&=[(\pi _{1},\pi _{2})\wedge (\sigma _{1}\wedge \rho _{1},\sigma _{2}\wedge \rho _{2})]\vee [(\pi _{1},\pi _{2})\wedge (\sigma _{1}\wedge \rho _{2},\sigma _{2}\wedge \rho _{1})] \text { by Prop.~2 (4)}\\&=[(\pi _{1}\wedge (\sigma _{1} \wedge \rho _{1}), \pi _{2} \wedge (\sigma _{2}\wedge \rho _{2}))\vee (\pi _{1}\wedge (\sigma _{2} \wedge \rho _{2}), \pi _{2} \wedge (\sigma _{1}\wedge \rho _{1})) ] \\&\quad \vee [(\pi _{1}{\wedge } (\sigma _{1} {\wedge } \rho _{2}), \pi _{2} \wedge (\sigma _{2}{\wedge } \rho _{1}))\vee (\pi _{1}{\wedge } (\sigma _{2} \wedge \rho _{1}), \pi _{2} {\wedge } (\sigma _{1}\wedge \rho _{2}) )]\text { by Prop.~2 (2)} \end{aligned}$$

By the inductive hypothesis *for both parts of the condition*, $\pi _{i} \wedge (\sigma _{j} \wedge \rho _{k})= (\pi _{i} \wedge \sigma _{j})\wedge \rho _{k}$ and $\pi _{i} \vee (\sigma _{j} \vee \rho _{k})= (\pi _{i} \vee \sigma _{j})\vee \rho _{k}$ for all $i,j,k \in \{1,2 \}$. We then get

$$\begin{aligned} \pi \wedge (\sigma \wedge \rho )&{=} [((\pi _{1}{\wedge } \sigma _{1}) {\wedge } \rho _{1}, (\pi _{2} \wedge \sigma _{2})\wedge \rho _{2})\vee ((\pi _{1}\wedge \sigma _{2}) \wedge \rho _{2}, (\pi _{2} \wedge \sigma _{1})\wedge \rho _{1})]\\&\quad \vee [((\pi _{1}{\wedge } \sigma _{1}) {\wedge } \rho _{2}, (\pi _{2} \wedge \sigma _{2})\wedge \rho _{1})\vee ((\pi _{1}{\wedge } \sigma _{2}) \wedge \rho _{1}, (\pi _{2} \wedge \sigma _{1})\wedge \rho _{2})]. \end{aligned}$$

By the inductive hypothesis for operator $\vee$ and by Condition 2, we can rearrange parentheses and swap the order of terms to obtain:

$$\begin{aligned} \pi \wedge (\sigma \wedge \rho )&=((\pi _{1}\wedge \sigma _{1}) \wedge \rho _{1}, (\pi _{2} \wedge \sigma _{2})\wedge \rho _{2})\vee [(\pi _{1}\wedge \sigma _{1}) \wedge \rho _{2}, (\pi _{2} \wedge \sigma _{2})\wedge \rho _{1})\\&\quad \vee ((\pi _{1}\wedge \sigma _{2}) \wedge \rho _{2}, (\pi _{2} \wedge \sigma _{1}){\wedge } \rho _{1}]\vee ((\pi _{1}\wedge \sigma _{2}) \wedge \rho _{1}, (\pi _{2} {\wedge } \sigma _{1}){\wedge } \rho _{2}). \end{aligned}$$

Dropping the brackets and viewing this expression as having four perfect phylogenies separated by the $\vee$ operator, we group the first two and the last two perfect phylogenies together and apply Proposition 2 (2) to each group. We get

$$\begin{aligned} \pi \wedge (\sigma \wedge \rho )&=[(\pi _{1}\wedge \sigma _{1},\pi _{2}\wedge \sigma _{2}) \wedge (\rho _{1},\rho _{2})]\\ {}&\quad \vee [(\pi _{1}\wedge \sigma _{2},\pi _{2}\wedge \sigma _{1}) \wedge (\rho _{1},\rho _{2})] \text { by Prop.~2 (2)}\\&=[(\pi _{1}\wedge \sigma _{1},\pi _{2}\wedge \sigma _{2}) \vee (\pi _{1}\wedge \sigma _{2},\pi _{2}\wedge \sigma _{1})] \wedge (\rho _{1},\rho _{2}) \text { by Prop.~2 (4)}\\&=(\pi \wedge \sigma )\wedge \rho \text { by Prop.~2 (2).} \end{aligned}$$

For the second part of the condition, suppose $\pi =(\pi _{1},\pi _{2}) \in \Pi _{n}$, $\sigma =(\sigma _{1},\sigma _{2}) \in \Pi _{n}$, and $\rho =(\rho _{1},\rho _{2})\in \Pi _{n}$ are three perfect phylogenies of size *n*. We consider four cases. First, suppose the three perfect phylogenies have mutually different subtree sizes—that is, $\{|\pi _1|,|\pi _2|\}$, $\{|\sigma _1|,|\sigma _2|\}$, and $\{|\rho _1|,|\rho _2|\}$ are mutually distinct. Then $\pi \vee \sigma = \pi \vee \rho = \sigma \vee \rho =(n)$ by Proposition 2 (3). We then have $\pi \vee (\sigma \vee \rho )=\pi \vee (n)=(n)= (n) \vee \rho = (\pi \vee \sigma ) \vee \rho$ by Definition 1 (4).

The same argument applies if it is merely assumed that $\sigma$ and $\rho$ have pairs of subtrees whose sizes differ, $\{|\sigma _1|,|\sigma _2|\} \ne \{|\rho _1|,|\rho _2|\})$. Then $\pi \vee (\sigma \vee \rho )=\pi \vee (n)=(n)=(\pi \vee \sigma )\vee \rho$ by Definition 1 (4) and Prop. 2 (3), where we have used the fact that $\sigma \vee \rho = (n)$ and $\sigma \le \pi \vee \sigma$, so that $(\pi \vee \sigma ) \vee \rho = (n)$.

If $\{|\sigma _1|,|\sigma _2|\} = \{|\rho _1|,|\rho _2|\})$ but $\{|\pi _1|,|\pi _2|\} \ne \{|\sigma _1|,|\sigma _2|\})$ and $\{|\pi _1|,|\pi _2|\} \ne \{|\rho _1|,|\rho _2|\})$, then $\pi \vee \sigma = (n)$. Because $\sigma \le \sigma \vee \rho$ and $\pi \vee \sigma = (n)$, $\pi \vee (\sigma \vee \rho ) = (n)$. Similarly, $(\pi \vee \sigma )\vee \rho = (n) \vee \rho = (n)$ by Definition 1 (4) and Prop. 2 (3).

It remains to consider the case in which at least a pair of subtrees, one each from $\pi$, $\sigma$ and $\rho$, have the same size, or $\{|\pi _1|,|\pi _2|\} = \{|\sigma _1|,|\sigma _2|\}) = \{|\rho _1|,|\rho _2|\})$. We have

23 $$\begin{aligned} \pi \vee (\sigma \vee \rho )&=(\pi _{1},\pi _{2}) \vee \left( (\sigma _{1},\sigma _{2}) \vee (\rho _{1},\rho _{2}) \right) \nonumber \\&=(\pi _{1},\pi _{2}) \vee [ (\sigma _{1} \vee \rho _{1}, \sigma _{2}\vee \rho _{2}) \wedge (\sigma _{1} \vee \rho _{2}, \sigma _{2}\vee \rho _{1})] \text { by Prop.~2 (3)} \nonumber \\&=[(\pi _{1},\pi _{2})\vee (\sigma _{1}\vee \rho _{1},\sigma _{2}\vee \rho _{2})]\nonumber \\ {}&\quad \wedge [(\pi _{1},\pi _{2})\vee (\sigma _{1}\vee \rho _{2},\sigma _{2}\vee \rho _{1})] \text { by Prop.~2 (4)} \nonumber \\&=[(\pi _{1}{\vee } (\sigma _{1} \vee \rho _{1}), \pi _{2} \vee (\sigma _{2}\vee \rho _{2})){\wedge }(\pi _{1}\vee (\sigma _{2} \vee \rho _{2}), \pi _{2} {\vee } (\sigma _{1}\vee \rho _{1})) ] \nonumber \\&\quad \wedge [(\pi _{1}\vee (\sigma _{1} \vee \rho _{2}), \pi _{2} \vee (\sigma _{2}\vee \rho _{1}))\wedge (\pi _{1}\vee (\sigma _{2} \vee \rho _{1}), \nonumber \\ {}&\quad \pi _{2} \vee (\sigma _{1}\vee \rho _{2})) ]\text { by Prop.~2 (3)} \nonumber \\&=((\pi _{1}\vee \sigma _{1}) \vee \rho _{1}, (\pi _{2} \vee \sigma _{2})\vee \rho _{2})\wedge ((\pi _{1}\vee \sigma _{1}) \vee \rho _{2}, (\pi _{2} \vee \sigma _{2})\vee \rho _{1}) \nonumber \\&\quad \wedge ((\pi _{1}\vee \sigma _{2}) \vee \rho _{2}, (\pi _{2} \vee \sigma _{1})\vee \rho _{1})\wedge ((\pi _{1}\vee \sigma _{2}) \vee \rho _{1},\nonumber \\ {}&\quad (\pi _{2} \vee \sigma _{1})\vee \rho _{2}) \text { by ind.~hypothesis} \nonumber \\&=[(\pi _{1}\vee \sigma _{1},\pi _{2}\vee \sigma _{2}) \vee (\rho _{1},\rho _{2})] \wedge [(\pi _{1}\vee \sigma _{2},\nonumber \\ {}&\quad \pi _{2}\vee \sigma _{1}) \vee (\rho _{1},\rho _{2})] \text { by Prop.~2 (3)} \nonumber \\&=[(\pi _{1}\vee \sigma _{1},\pi _{2}\vee \sigma _{2}) \wedge (\pi _{1}\vee \sigma _{2},\pi _{2}\vee \sigma _{1})] \vee (\rho _{1},\rho _{2}) \text { by Prop.~2 (4)}\nonumber \\&=(\pi \vee \sigma )\vee \rho \text { by Prop.~2 (3).} \end{aligned}$$

Note that this derivation includes the case of shared subtrees at the root, in which it is not only the sizes of the subtrees that are the same, but the subtrees themselves. For example, suppose $\pi =(\pi _{1},\pi _{2})$ and $\sigma =(\pi _{1},\sigma _{1})$. By Proposition 2 (3), we have

$$\begin{aligned} \pi \vee \sigma&= (\pi _{1},\pi _{2})\vee (\pi _{1},\sigma _{1})= (\pi _{1},\pi _{2}\vee \sigma _{1}). \end{aligned}$$

However, we will show that we can replace the previous equality by the extended expression:

24 $$\begin{aligned} \pi \vee \sigma&= (\pi _{1}\vee \pi _{1},\pi _{2}\vee \sigma _{1}) \wedge (\pi _{1}\vee \sigma _{1},\pi _{1}\vee \pi _{2}), \end{aligned}$$

and then the previous derivation remains unchanged. To prove this assertion, we have:

$$\begin{aligned} \pi _{2}\vee \sigma _{1}&=(\pi _{2} \vee (\pi _{1}\wedge \pi _{2})) \vee \sigma _{1} \quad \text { by Condition 4}\\&=(\pi _{2} \vee \sigma _{1}) \vee (\pi _{1}\wedge \pi _{2}) \quad \text { by the inductive hypothesis and Condition 2}\\&=\pi _{2}\vee [(\sigma _{1} \wedge \pi _{1})\vee \sigma _{1} ]\vee (\pi _{1}\wedge \pi _{2}) \quad \text { by Conditions 2 and 4}\\&=[\pi _{2}\vee (\sigma _{1} \wedge \pi _{1})]\vee [\sigma _{1}\vee (\pi _{1}\wedge \pi _{2})] \quad \text { by the inductive hypothesis.} \end{aligned}$$

Then

25 $$\begin{aligned} \pi \vee \sigma&= (\pi _{1},\pi _{2}\vee \sigma _{1}) \nonumber \\&=(\pi _{1},[\pi _{2}\vee (\sigma _{1} \wedge \pi _{1})]\vee [\sigma _{1}\vee (\pi _{1}\wedge \pi _{2})]) \nonumber \\&=(\pi _{1},\pi _{2}\vee (\sigma _{1}\wedge \pi _{1}))\vee (\pi _{1},\sigma _{1}\vee (\pi _{1}\wedge \pi _{2})) \quad \text { by Prop.~2 (3)} \nonumber \\&=(\pi _{1},(\pi _{2}\vee \sigma _{1}) \wedge (\pi _{2}\vee \pi _{1})) \vee (\pi _{1},(\sigma _{1}\vee \pi _{1})\wedge (\sigma _{1}\vee \pi _{2})) \quad \text { by Prop.~2 (4).} \end{aligned}$$

By Condition 4 and Proposition 2 (3) we have

$$\begin{aligned} \pi _{1}&=\pi _{1} \vee (\pi _{1}\wedge \sigma _{1})=(\pi _{1} \vee \pi _{1}) \wedge ( \pi _{1}\vee \sigma _{1}), \end{aligned}$$

and

$$\begin{aligned} \pi _{1}&=\pi _{1} \vee (\pi _{1}\wedge \pi _{2})= (\pi _{1} \vee \pi _{1})\wedge (\pi _{1}\vee \pi _{2}). \end{aligned}$$

Replacing the first $\pi _{1}$ in the first pair of Eq. 25 by $(\pi _{1} \vee \pi _{1}) \wedge ( \pi _{1}\vee \sigma _{1})$, and the first $\pi _{1}$ in the second pair of Eq. 25 by $(\pi _{1} \vee \pi _{1}) \wedge ( \pi _{1}\vee \pi _{2})$, we get

$$\begin{aligned} \pi \vee \sigma&=((\pi _{1} \vee \pi _{1}) \wedge ( \pi _{1}\vee \sigma _{1}),(\pi _{2}\vee \sigma _{1}) \wedge (\pi _{2}\vee \pi _{1})) \vee ((\pi _{1} \vee \pi _{1})\\ {}&\quad \wedge ( \pi _{1}\vee \pi _{2}),(\sigma _{1}\vee \pi _{1})\wedge (\sigma _{1}\vee \pi _{2}))\\&=(\pi _{1}\vee \pi _{1},\pi _{2}\vee \sigma _{1}) \wedge (\pi _{1}\vee \sigma _{1},\pi _{1}\vee \pi _{2})\quad \text { by Prop.~2 (2).} \end{aligned}$$

Thus, Eq. 24 holds, so that Eq. 23 holds for the case in which subtrees are shared at the root.

## Appendix: Proof of Proposition 2

1. Let $\pi _{1}=(n_{1},n_{2})$ and $\pi _{2}=(n_{3},n_{4})$ be two perfect phylogenies in $\Pi _{n}$ with $n_{1}+n_{2}=n_{3}+n_{4}=n$. Then
  $$\begin{aligned} \pi _{1} \vee \pi _{2}=(n_{1},n_{2}) \vee (n_{3},n_{4})={\left\{ \begin{array}{ll} (n_{1},n_{2}) &{}\quad \text { if } n_{1}=n_{3} \text { or } n_{1}=n_{4}\\ (n) &{}\quad \text { otherwise}. \\ \end{array}\right. } \end{aligned}$$

### Proof

(a) If $n_{1}=n_{3}$ (or $n_{1}=n_{4}$), then $n_{2}=n_{4}$ (or $n_{2}=n_{3}$) since $n_{1}+n_{2}=n_{3}+n_{4}$ hence $\pi _{1}=\pi _{2}$. This in turn implies that $\pi _{1}\le \pi _{2}$ and therefore $\pi _{1}\vee \pi _{2}=\pi _{2}=\pi _{1}$.

(b) If $n_{1}\ne n_{3}$ and $n_{1}\ne n_{4}$, then by definition $\pi _{1}\vee \pi _{2}$ is the smallest perfect phylogeny that is refined by both $\pi _{1}$ and $\pi _{2}$. Clearly (*n*) is refined by both $\pi _{1}$ and $\pi _{2}$ and this is the smallest since (*n*) is directly obtained by collapsing the pair of pendant edges of $\pi _{1}$ and $\pi _{2}$. $\square$

2. For all $\pi _{1}$, $\pi _{2}$, $\pi _{3}$, $\pi _{4}$ with $(\pi _{1},\pi _{2}) \in \Pi _{n}$ and $(\pi _{3},\pi _{4}) \in \Pi _{n}$,
  $$\begin{aligned} (\pi _{1},\pi _{2}) \wedge (\pi _{3},\pi _{4})= (\pi _{1}\wedge \pi _{3}, \pi _{2} \wedge \pi _{4}) \vee (\pi _{1}\wedge \pi _{4}, \pi _{2} \wedge \pi _{3}), \end{aligned}$$
  with the convention that $(\pi ,\emptyset )=\emptyset$.

### Proof

The meet of the two perfect phylogenies is the largest perfect phylogeny that refines both $(\pi _{1},\pi _{2})$ and $(\pi _{3},\pi _{4})$, that is, the largest among $(\pi _{1}\wedge \pi _{3},\pi _{2}\wedge \pi _{4})$ and $(\pi _{1}\wedge \pi _{4},\pi _{2}\wedge \pi _{3})$, and the largest corresponds to their join. $\square$

3. For all $\pi _{1}$, $\pi _{2}$, $\pi _{3}$, $\pi _{4}$ with $(\pi _{1},\pi _{2}) \in \Pi _{n}$ and $(\pi _{3},\pi _{4}) \in \Pi _{n}$, $\pi _{i}\in \Pi _{n_{i}}$ for $i=1,2,3,4$,
  $$\begin{aligned}&(\pi _{1},\pi _{2}) \vee (\pi _{3},\pi _{4})\\&\quad ={\left\{ \begin{array}{ll} (n) &{} \text { if } n_{1}\ne n_{3} \text { and } n_{1} \ne n_{4}\\ (\pi _{1}, \pi _{2} \vee \pi _{4}) &{} \text { if } \pi _{1}=\pi _{3}\\ (\pi _{1}, \pi _{2} \vee \pi _{3}) &{} \text { if } \pi _{1}=\pi _{4}\\ (\pi _{2}, \pi _{1} \vee \pi _{4}) &{} \text { if } \pi _{2}=\pi _{3}\\ (\pi _{2}, \pi _{1} \vee \pi _{3}) &{} \text { if } \pi _{2}=\pi _{4}\\ (\pi _{1}\vee \pi _{3}, \pi _{2} \vee \pi _{4}) \wedge (\pi _{1}\vee \pi _{4}, \pi _{2} \vee \pi _{3}) &{} \text { otherwise}, \end{array}\right. } \end{aligned}$$
  with the convention that $(\pi ,\emptyset )=\emptyset$.

### Proof

The join of two perfect phylogenies is the smallest perfect phylogeny that is refined by both $(\pi _{1},\pi _{2})$ and $(\pi _{3},\pi _{4})$. (a) If $n_{1}\ne n_{3}$ and $n_{1}\ne n_{4}$, then no subtree of $\pi _{i}$, $i=1,\ldots ,4$ can be refined from any other by Definition 1 (6), therefore the join corresponds to the perfect phylogeny obtained by collapsing the pair of pendant edges of $(\pi _{1},\pi _{2})$ and $(\pi _{3},\pi _{4})$ which in both cases correspond to (*n*). (b) If one cross-pair of subtrees is identical, for example, $\pi _{1}=\pi _{3}$, then clearly $\pi _{1}\vee \pi _{3}=\pi _{1}$ since $\pi _{1}$ is refined by both $\pi _{1}$ and $\pi _{3}$, therefore the perfect phylogeny that is refined by both $(\pi _{1},\pi _{2})$ and $(\pi _{1},\pi _{4})$ is $(\pi _{1},\pi _{2}\vee \pi _{4})$. (c) Otherwise, the join corresponds to the meet of the two perfect phylogenies that join two different cross-pairs from $(\pi _{1},\pi _{2})$ and $(\pi _{3},\pi _{4})$. $\square$

4. For all $$\pi _{1},\pi _{2}, \pi _{3} \in \Pi _{n},$$
  $$\begin{aligned} \pi _{1} \wedge (\pi _{2} \vee \pi _{3})=(\pi _{1} \wedge \pi _{2}) \vee (\pi _{1} \wedge \pi _{3}), \end{aligned}$$
  and
  $$\begin{aligned} \pi _{1} \vee (\pi _{2} \wedge \pi _{3})=(\pi _{1} \vee \pi _{2}) \wedge (\pi _{1} \vee \pi _{3}). \end{aligned}$$

### Proof

In the first case, $\pi _{1} \wedge (\pi _{2}\vee \pi _{3})$ is the largest perfect phylogeny that refines both $\pi _{1}$ and $(\pi _{2},\pi _{3})$. This perfect phylogeny then refines both $\pi _{1}$ and $\pi _{2}$ or both $\pi _{1}$ and $\pi _{3}$, that is, the largest of $(\pi _{1},\pi _{2})$ and $(\pi _{1},\pi _{3})$ and this largest perfect phylogeny corresponds to their join. In the second case, $\pi _{1} \vee (\pi _{2} \wedge \pi _{3})$ is the smallest perfect phylogeny that is refined by both $\pi _{1}$ and $(\pi _{2}\vee \pi _{3})$, that is, refined by both $\pi _{1}$ and $\pi _{2}$, and by $\pi _{1}$ and $\pi _{3}$ and this corresponds to the meet of the perfect phylogeny that is refined by $\pi _{1}$ and $\pi _{2}$ and the perfect phylogeny that is refined by $\pi _{1}$ and $\pi _{3}$. $\square$

5. Let $\pi , \sigma \in \Pi _{n}$ be two perfect phylogenies that are not comparable. There exist unique $\gamma ,\rho \in (\Pi _{n} \cup \{\emptyset \}) {\setminus } \{\pi ,\sigma \}$ such that
  $$\begin{aligned} \pi \wedge \sigma = \gamma , \quad \pi \vee \gamma = \pi , \quad \text { and }\quad \sigma \vee \gamma =\sigma , \end{aligned}$$
  and
  $$\begin{aligned}\pi \vee \sigma = \rho , \quad \pi \wedge \rho = \pi , \quad \text { and }\quad \sigma \wedge \rho =\sigma . \end{aligned}$$

### Proof

The meet of two incomparable perfect phylogenies is the largest perfect phylogeny that refines both $\pi$ and $\sigma$. If $\pi$ or $\sigma$ or both are perfect phylogenies with all tips labeled 1, for example ((1, 1), 1) then the only refinement of $\pi$ and $\sigma$ is $\emptyset$ and the result holds by Definition 1. Otherwise a refinement of each perfect phylogeny can be obtained sequentially by branching any tip with label greater than 1, until a common perfect phylogeny is reached or until two perfect phylogenies with all tips labeled 1 are reached. If a common perfect phylogeny $\gamma$ is reached we then have $\gamma \vee \pi =\pi$ and $\gamma \vee \sigma =\sigma$ and the result holds. If no common perfect phylogeny is reached then $\gamma =\emptyset$ and the result holds. Similarly, the join of two incomparable perfect phylogenies is the smallest perfect phylogeny refined by both $\pi$ and $\sigma$. Since $\pi$ and $\sigma$ are not comparable then $\pi$ nor $\sigma$ are (*n*), therefore we can sequentially collapse pairs of pendant edges of $\pi$ and $\sigma$ until a common perfect phylogeny is reached or (*n*) is reached. In both cases, the result holds. $\square$
